# Whole-genome sequencing expands diagnostic utility and improves clinical management in paediatric medicine

**DOI:** 10.1038/npjgenmed.2015.12

**Published:** 2016-01-13

**Authors:** Dimitri J Stavropoulos, Daniele Merico, Rebekah Jobling, Sarah Bowdin, Nasim Monfared, Bhooma Thiruvahindrapuram, Thomas Nalpathamkalam, Giovanna Pellecchia, Ryan K C Yuen, Michael J Szego, Robin Z Hayeems, Randi Zlotnik Shaul, Michael Brudno, Marta Girdea, Brendan Frey, Babak Alipanahi, Sohnee Ahmed, Riyana Babul-Hirji, Ramses Badilla Porras, Melissa T Carter, Lauren Chad, Ayeshah Chaudhry, David Chitayat, Soghra Jougheh Doust, Cheryl Cytrynbaum, Lucie Dupuis, Resham Ejaz, Leona Fishman, Andrea Guerin, Bita Hashemi, Mayada Helal, Stacy Hewson, Michal Inbar-Feigenberg, Peter Kannu, Natalya Karp, Raymond H Kim, Jonathan Kronick, Eriskay Liston, Heather MacDonald, Saadet Mercimek-Mahmutoglu, Roberto Mendoza-Londono, Enas Nasr, Graeme Nimmo, Nicole Parkinson, Nada Quercia, Julian Raiman, Maian Roifman, Andreas Schulze, Andrea Shugar, Cheryl Shuman, Pierre Sinajon, Komudi Siriwardena, Rosanna Weksberg, Grace Yoon, Chris Carew, Raith Erickson, Richard A Leach, Robert Klein, Peter N Ray, M Stephen Meyn, Stephen W Scherer, Ronald D Cohn, Christian R Marshall

**Affiliations:** 1 Genome Diagnostics, Department of Paediatric Laboratory Medicine, The Hospital for Sick Children, Toronto, ON, Canada; 2 Department of Laboratory Medicine and Pathobiology, University of Toronto, Toronto, ON, Canada; 3 The Centre for Applied Genomics, The Hospital for Sick Children, Toronto, ON, Canada; 4 Genetics and Genome Biology Program, The Hospital for Sick Children, Toronto, ON, Canada; 5 Division of Clinical and Metabolic Genetics, The Hospital for Sick Children, Toronto, ON, Canada; 6 Centre for Genetic Medicine, The Hospital for Sick Children, Toronto, ON, Canada; 7 Department of Paediatrics, University of Toronto, Toronto, ON, Canada; 8 Joint Centre for Bioethics, University of Toronto, Toronto, ON, Canada; 9 Department of Family and Community Medicine and Clinical Public Health Division, University of Toronto, Toronto, ON, Canada; 10 Child Health Evaluative Sciences, The Hospital for Sick Children, Toronto, Ontario, Canada; 11 Department of Bioethics, The Hospital for Sick Children, Toronto, ON, Canada; 12 Centre for Computational Medicine, The Hospital for Sick Children, Toronto, ON, Canada; 13 Department of Computer Science, University of Toronto, Toronto, ON, Canada; 14 Department of Electrical and Computer Engineering and Donnelly Centre for Cellular and Biomolecular Research, University of Toronto, Toronto, Ontario, Canada; 15 Department of Molecular Genetics, University of Toronto, Toronto, Ontario, Canada; 16 Prenatal Diagnosis and Medical Genetics Program, Mount Sinai Hospital, Toronto, ON, Canada; 17 Department of Paediatric Laboratory Medicine, The Hospital for Sick Children, Toronto, ON, Canada; 18 Department of Ophthalmology and Vision Sciences, The Hospital for Sick Children, Toronto, ON, Canada; 19 Complete Genomics Inc, Mountain View, CA, USA

## Abstract

The standard of care for first-tier clinical investigation of the aetiology of congenital malformations and neurodevelopmental disorders is chromosome microarray analysis (CMA) for copy-number variations (CNVs), often followed by gene(s)-specific sequencing searching for smaller insertion–deletions (indels) and single-nucleotide variant (SNV) mutations. Whole-genome sequencing (WGS) has the potential to capture all classes of genetic variation in one experiment; however, the diagnostic yield for mutation detection of WGS compared to CMA, and other tests, needs to be established. In a prospective study we utilised WGS and comprehensive medical annotation to assess 100 patients referred to a paediatric genetics service and compared the diagnostic yield versus standard genetic testing. WGS identified genetic variants meeting clinical diagnostic criteria in 34% of cases, representing a fourfold increase in diagnostic rate over CMA (8%*; P* value=1.42E−05) alone and more than twofold increase in CMA plus targeted gene sequencing (13%; *P* value=0.0009). WGS identified all rare clinically significant CNVs that were detected by CMA. In 26 patients, WGS revealed indel and missense mutations presenting in a dominant (63%) or a recessive (37%) manner. We found four subjects with mutations in at least two genes associated with distinct genetic disorders, including two cases harbouring a pathogenic CNV and SNV. When considering medically actionable secondary findings in addition to primary WGS findings, 38% of patients would benefit from genetic counselling. Clinical implementation of WGS as a primary test will provide a higher diagnostic yield than conventional genetic testing and potentially reduce the time required to reach a genetic diagnosis.

## Introduction

Congenital anomalies are a leading cause of infant mortality, and developmental disabilities have profound adverse effects on children, their families, health care systems and societies.^1^ Collectively, children with these disorders comprise 5–10% of the general population^2^ and encompass the largest group referred for genetic evaluation. These numbers are significant given that 50–80% of the resources used to manage diseases in full-service paediatric inpatient facilities have a recognised genetic component.^3,4^

The ability to provide optimal clinical management for these individuals is dependent on identifying the underlying genetic cause in order to determine prognosis, guide treatment and institute appropriate surveillance and prevention programmes.^5^ However, it is often difficult to achieve a definitive genetic diagnosis, for example, in children with developmental delay, autism and/or congenital anomalies because their phenotypic features are often nonspecific and the differential diagnosis can include hundreds of rare genetic disorders.^3,4^

The development of chromosome microarray analysis (CMA) revealed that copy-number variation (CNV) is a major aetiology for congenital malformations and neurodevelopmental delay. CMA has now become the first-line diagnostic test for these disorders, achieving greater than a twofold increase in diagnostic yield compared with G-band karyotype analysis.^6,7^ Despite these successes, 80–85% of patients do not reach a diagnosis by CMA.^7^ Consequently, physicians often supplement CMA with targeted testing by sequence analysis of known disease-associated genes or gene panels. This hypothesis-driven approach, which relies on the ability to recognise the most likely disorder associated with the presenting symptoms, often fails to reach a diagnosis.

Recent improvement in cost and accuracy of whole-exome sequencing (WES) has made it feasible to investigate all known coding genes for sequence-level mutations. Current estimates show clinical WES provides a diagnostic yield of ~25% for patients affected with neurological disorders and/or congenital anomalies.^8,9^ Although larger clinically relevant CNVs may be detected through WES,^10^ the lack of sensitivity and reliability to detect smaller CNVs (<100 kb) and insertion–deletions (indels), as well as complex structural variations (SVs) has precluded its implementation as a single clinical test able to capture all potential disease-causing genetic variants.^8,9^ As such, sequential testing using CMA, WES or targeted gene-based testing often occurs.

Whole-genome sequencing (WGS) has the potential to identify nearly all forms of genetic variation.^11^ Several studies have demonstrated the advantages of WGS for mutation detection^12–15^ and WGS analyses of paediatric populations has shown identification of clinically relevant variants in ~40% of those with autism^15^ and ~60% of those with intellectual disability.^16,17^ These observations indicate that WGS is poised to have tremendous impact for paediatric patients where CMA is currently a standard first-line diagnostic evaluation. However, challenges such as cost, processing, clinical interpretation and storage of vast amounts of data exist^18^ and evidence is required to demonstrate the diagnostic utility of WGS.^19^ Here we performed WGS on 100 consecutive children referred for CMA by clinical geneticists and examined the diagnostic yield of WGS compared with conventional molecular testing.

## Results

### Cohort enrolment and description

We recruited 100 consecutive paediatric patients (57% males) <18 years of age (mean 5.5 years; Supplementary Table 1) who met criteria for CMA, and WGS was offered in parallel with clinical CMA testing. These 100 cases came from 201 families that were approached (95 declining participation and 6 undecided). Eight percent of families reported consanguinity. Of those families that enroled, 26% opted out of receiving information about secondary findings related to medically actionable adult onset disorders.^20,21^ Patients displayed a wide array of symptoms described by 453 unique HPO terms across the cohort, with 57% having at least one term associated with developmental delay (Supplementary Table 2; Supplementary Figures 2 and 3). The most commonly observed phenotypes were abnormalities of the nervous system (77%), skeletal system (68%), growth (44%), eye (34%), cardiovascular (32%) and musculature (27%; Supplementary Table 3). Aside from CMA, an average of two additional genetic diagnostic tests were ordered at the time of WGS analysis (Range 1–13 tests; Supplementary Table 4).

### Whole-genome sequencing

WGS yielded an average depth coverage of 51.8× with 99% of the mapped sequence at >10-fold representation (Supplementary Table 5). Different WGS technologies will vary in the number of variants identified. The WGS platform used in this study generated a total of 3.3 to 4.3 million high-quality variants per sample. On average, WGS identified >3.5 million SNVs, 248 CNVs using read depth method and 1,604 structural variations (tandem duplications and deletions) using abnormal junction and discordant mate-pair clusters (Supplementary Methods and Supplementary Tables 6 and 7). Filtering for coding variants, we detected an average of 20,014 exonic and splicing variants per individual. As expected, CNVs identified by read depth (median size 10 kb) were larger than the SVs detected by split read or mate-pair mapping (median size 495 bp). Approximately 28% of the CNVs and 2.0% of the SVs impacted gene coding exons. WGS data are deposited in the European Genome-phenome Archive (www.ebi.ac.uk/ega/) under accession number EGAS00001001623.

### Variant analysis and molecular diagnosis using WGS

We developed a pipeline to prioritise WGS variants (SNVs and Indels) of clinical significance, interrogating on average 498 rare damaging events per genome parsed into categories based on mode of inheritance (Supplementary Methods and Supplementary Figure 1). Rare CNVs and SVs were analysed for pathogenicity according to established methods^22,23^ and candidates were discussed with the referring clinician to assess whether the variant(s) were pathogenic or related to the phenotype and therefore considered to be clinically relevant.

Overall, we identified and returned 38 variants that were related to the primary indication providing a molecular diagnosis for 34 individuals (34%; 95% confidence interval (CI) 25–44%) (Table 1). Of the positive diagnoses, 8 (8%) individuals harboured a pathogenic CNV (Table 2), whereas 28 (28%) carried sequence-level variants that were diagnostic (Table 3). The majority of sequence-level variants were autosomal dominant (63%) compared to recessive (37%) with no X-linked forms found. All pathogenic CNVs detected by WGS were confirmed with CMA and all diagnostic sequence-level variants were confirmed with Sanger sequencing. The diagnostic rate for those with developmental delay was higher than average 38.6% (22/57) and was lowest (15.3%) in those presenting with connective tissue disorders (Supplementary Figure 4 and Supplementary Figure 5). Combining CNVs and SNVs, we confirmed *de novo* disease-causing variants in 15% of this case cohort.

We observed that 4% of cases had pathogenic variants at two distinct disease loci leading to a composite phenotype.^8,9^ This is likely an underestimate given that in several patients the diagnostic findings only explained part of the clinical features (see patients (1006, 1040, 1062, 1070, 1090 in Tables 2 and 3). Interestingly, two of our patients harboured a pathogenic CNV and SNV (1066 and 1102 in Table 2). For several cases, the genomic diagnosis impacted clinical management and the identification of at-risk relatives (see Table 3 and Supplementary Table 8 for patient case examples).

### Secondary findings

Although our focus was the investigation of the diagnostic yield of WGS for the primary presenting clinical symptoms, we also examined the 56 genes listed in the 2013 American College of Medical Genetics and Genomics (ACMG) published guidelines for incidental findings^20^ and identified 7 variants as potentially medically actionable and appropriate for return (Table 4). Three of these seven patients (1027, 1040, and 1078) also had primary diagnostic variants.

### Comparison of CNV calling from WGS versus CMA

An important consideration in the evaluation of WGS as a clinical test is the sensitivity in detecting clinically relevant CNVs. We examined the characteristics of CNVs detected by WGS and CMA (Supplementary Table 7 and Supplementary Figure 7). CMA identified an average of six CNVs per patient including nine pathogenic CNVs in eight individuals (ranging in size from 337 kb to 92 Mb). All of the reported pathogenic changes were detected by WGS (Table 2). We evaluated concordance of the results obtained from the clinical microarray analysis with CNVs obtained from the WGS. CMA detected a total of 578 variants, of which 52% were detected by WGS consistent with published findings.^17,24^

The WGS data afforded several advantages over CMA for CNV detection. First, the resolution of WGS is greater than CMA, typically detecting >1,500 unbalanced changes that cannot be found using CMA. The majority of these are small and intergenic but many impact exons and may therefore be medically relevant. Although we did not find a plausible diagnosis from one of the variants beyond the resolution of CMA, we did detect carriers with clinically relevant exonic deletions in genes associated with autosomal recessive disorders (e.g., deletion of exons 7–8 of CLN3; neuronal ceroid lipofuscinosis-3; Supplementary Table 9). An additional advantage of WGS is demonstrated by using paired-end sequencing to obtain breakpoint resolution and allele specific CNVs. For example, both the WGS read depth and CMA detected a 300-kb duplication of uncertain significance (VUS) at 9p24.3 in one patient, but split read mapping revealed it to be a 503,479-bp tandem duplication on one allele overlapping a 235,071-bp deletion on the other allele (data not shown).

### Comparison of diagnostic yield of WGS versus CMA and standard genetic testing

The total diagnostic rate from standard testing (CMA plus targeted gene sequencing) was less than half of WGS (13% vs. 34%*; P* value=0.0009; Table 1). Of the targeted sequence tests ordered, 17 cases were negative for a diagnosis found through WGS, including 11 panel tests, highlighting the limitation of using a hypothesis-driven approach for this cohort. In the majority of these cases, the causative gene was not included in the panel testing; however, in one case, clinical panel testing failed to detect a large 25-bp pathogenic indel in *CBL* that was detected through WGS (Supplementary Figure 6 and Case 1050 in Supplementary Table 8). In two cases, targeted genetic tests led to a clinical diagnosis that could not be detected by WGS in the current study design, including microsatellite analysis of parents and offspring for UPD14 (heterodisomy) and a methylation test for Russell–Silver syndrome (RSS).

## Discussion

Here we provide data that show WGS exceeds other technology platforms in ability to detect genetic variants involved in childhood disease. Specifically, in our design we achieved a diagnostic yield of 34% when testing an unselected paediatric population that was undergoing CMA as the current standard first-tier genetic test followed by traditional gene/panel testing protocols. These results indicate WGS provides a fourfold increase in molecular diagnosis over CMA alone (8%) and a greater than twofold increase when all genetic testing protocols (>10 tests in some cases) are considered (13%).

The cohort we investigated was clinically heterogeneous with ~57% presenting with developmental delay as a primary clinical feature. Consistent with previous data,^8,9,25^ the majority (63%) of our sequence-level diagnoses were autosomal dominant. Combining CNVs and SNVs, just under half (14/34) of the diagnoses were *de novo* mutations giving a minimum overall spontaneous mutation rate of 14%. For autosomal recessive transmission, we confirmed molecular diagnoses from compound heterozygous variants in only 2/100 patients, which is less than reported previously^8,9,25^ and may be reflective of our study design in only sequencing the proband. The majority of our AR diagnoses arose from homozygous variants from consanguineous unions (reported in 8% of cases in this cohort). Similar to other studies,^8,9^ 4% of cases had more than one locus involved, contributing to a complex phenotype including two individuals with a pathogenic CNV and SNV. In an additional five cases, a pathogenic variant was found that only accounted for part of the phenotype, indicating that upwards of 9% of individuals in this cohort may have more than one genetic disorder (see Tables 2 and 3).

Our results demonstrate that the increased diagnostic utility of WGS can have a significant impact on clinical care and management that goes beyond genetic counselling (Tables 2 and 3). Specifically, the rapid diagnosis of mutations in the *CBL* gene in patient 1050 has important implications for the surveillance of juvenile myelomonocytic leukaemia, which is critical for patient management and survival. In the case of patient 1049, the clinical phenotype was suggestive of a connective tissue disorder with additional features suggestive of *NF1*. However, WGS revealed a diagnosis of Sotos syndrome, which subsequently changed clinical management of the patient as the disease trajectory and requirements for surveillance are inherently different for this disorder. Similarly, detection and confirmation of mutations in genes such as *PIK3R1, EXT2, PIK3R2, NGLY1, KAT6B* and *COL4A1* provides indications for monitoring of disease-specific secondary complications that ultimately lead to improvement of the patients’ quality of life, and at times can have a critical impact on survival.

One of the current challenges facing health care providers is determining the most effective utilisation of CMA versus sequencing gene panels versus WES in patients with developmental disorders and/or congenital anomalies. A recent study using a comprehensive genotype-driven approach in children with developmental delay achieved a diagnostic yield of 31% using WES and CMA.^25^ The physicians in our study ordered an average of three genetic tests (CMA plus two targeted genetic tests) per patient guided by clinical features, which yielded a diagnostic rate half of that achieved by WGS. Importantly, hypothesis-free WGS significantly outperformed targeted testing of candidate genes in our cohort: in 17/22 cases, the diagnostic sequence-level variant found by WGS was not in a gene targeted by hypothesis-driven testing. The number of prior genetic investigations for the illustrative cases (Supplementary Table 8) ranged between three and six tests at a total cost of $3,325–5,280 and would be largely representative of this type of cohort seen at our hospital. Estimates of others have shown even higher costs of negative testing ($19,100) in similar cohorts and have demonstrated the cost of using genomic sequencing to be ~$3,000 per individual.^26^ Although full economic evaluations are required, our data align with previous studies where WGS enabled the use of a single diagnostic test in a heterogeneous clinical cohort. In turn, this approach is likely to reduce the number of genetic investigations and potentially the time to diagnosis, ultimately acting as a more cost effective approach.^26,27^

Although our study design was prospective, there are several factors that may have influenced our estimation of the diagnostic yield of WGS. The diagnostic laboratory at The Hospital for Sick Children (Toronto, ON, Canada) receives ~600 CMA requests from clinical genetics per year and typically achieves a diagnostic yield of 12% (95% CI 9.6–14.9%). The yield of CMA in the current study was slightly lower (8%) than typically observed, but was similar in those individuals that were approached and declined WGS. For the 101 families who were approached and declined participation there were two predominant reasons. First, ~35% of families were uncomfortable with secondary findings as it pertains to obtaining life or employment insurance as Canada does not currently have a nondiscrimination law. Second, 35% of individuals declined since they felt overwhelmed with the current medical complexity of their child with most of these patients stemming from the NICU population. Although there is a chance that our cohort had an ascertainment bias, enrolment demographics indicate that this cohort is typical of those submitted for CMA through clinical genetics over the same timeframe.

One of the unique aspects in our study was the direct interaction with the referring clinician to adjudicate whether prioritised variants were medically relevant. This type of iterative genotype–phenotype comparison is valuable in the interpretation of a genome but is not amenable to large-scale implementation of WGS diagnostic testing that would be done at a reference laboratory. However, the phenotypic description submitted by the referring physician via PhenoTips (www.phenotips.org) was sufficient to confidently identify the clinically significant variants in the vast majority of diagnoses, without the need for further consultation with the physician. We estimate that approximately two diagnostic variants benefited from direct clinician consultation after the genomic data were analysed.

In addition, our design did not allow the prioritisation of *de novo* variants since we did not perform WGS on parents. WES-based studies have shown that the diagnostic yield is higher using a trio design.^9,18^ Similarly, although WGS allows the detection of large stretches of homozygosity due to uniparental isodisomy, we would need parental genotypes to detect heterodisomy (e.g., UPD14 in case 1068). Moreover, our analysis was restricted mainly to exonic variants due to the inability to clinically interpret the majority of intronic and intergenic variants. The impact of common risk variants will also need to be considered as methods for complex statistical modelling improve. Finally, we did not detect any pathogenic CNVs beyond the resolution of CMA that led to a diagnosis in our cohort, but did find some individuals to be carriers of deletions affecting genes associated with autosomal recessive disorders (Supplementary Table 9).

Genetics have long been known to have a major role in child health. McCandless *et al.* ^3^ found that, taken together, rare genetic variants and common genetic factors significantly contributed to the illnesses of 71% of hospitalised children. However, our ability to achieve molecular diagnoses for children with genetic disorders has historically been quite limited. Our results indicate that WGS can now be deployed advantageously as a first-tier molecular test in those individuals with developmental delay and/or congenital abnormalities, as we identified medically actionable diagnostic variants and secondary variants in 38% of children undergoing clinical CMA, and achieved a diagnosis in one-third of the children. As comparison datasets increase in size and methods for annotating the vast non-genic segment (99%) of the genome improve, so will the utility of WGS in diagnosis, management and surveillance of paediatric genetic conditions.

## Materials and methods

### Patient selection/study cohort and phenotype collection

**Figure 1:**
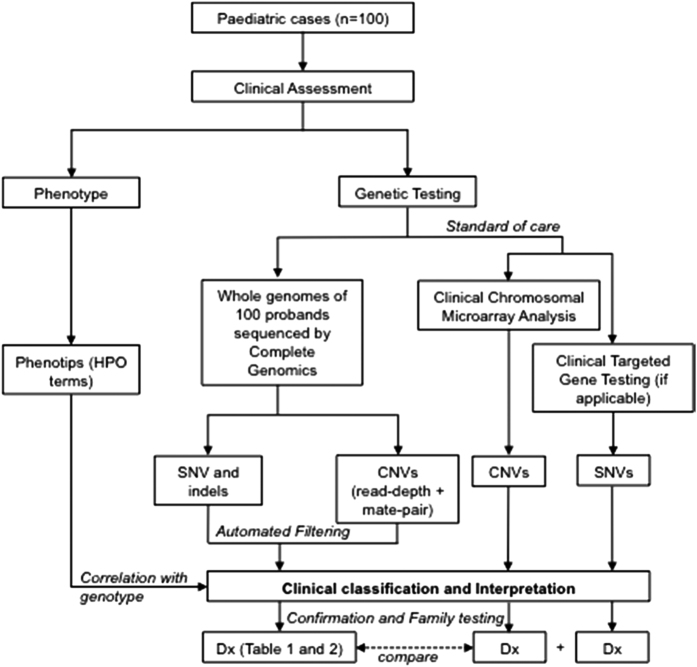
Overview of study design comparing the diagnostic yield of whole-genome sequencing compared with standard of care genetic testing. CMA, chromosomal microarray analysis; SNV, single-nucleotide variant; CNV, copy-number variant; HPO, Human Phenotype Ontology; Dx, diagnostic.

We developed a workflow to recruit patients and test the diagnostic utility of WGS in the routine clinical care of children and their families (Figure 1). Patients were recruited in a prospective manner from the Division of Clinical and Metabolic Genetics at Hospital for Sick Children over a 9-month period (September 2013 to May 2014). Patients and their parents were eligible to participate in the study if the patient met standard clinical criteria for CMA analysis. This included children with two or more structural malformations (major or minor); or unexplained developmental delay/intellectual disability with or without additional clinical features. We also required both parents be available for testing and due to the complexity of the tests, that both parents were fluent in English. DNA was extracted from peripheral blood. This study was approved by the Research Ethics Board at The Hospital for Sick Children and informed consent was obtained from all participants.

### Phenotype collection

We used PhenoTips^28^ (www.phenotips.org) to capture and record phenotypic data after thorough examination by a clinical geneticist. PhenoTips is an open source software program for collecting and analysing phenotypic information for patients with genetic disorders. The software combines an easy-to-use web browser interface with a standardized database back end. Phenotypic information is collected and represented using the Human Phenotype Ontology.^29^ Collected data include demographics, medical history, family history, physical and laboratory measurements, physical findings, and additional notes. Importantly, phenotypic data for each patient were accessible in a standardized format by laboratory geneticists and clinical geneticists performing variant interpretation.

### Chromosomal microarray analysis

All cases underwent chromosomal microarray analysis (CMA) using the 4×180 K Cytosure ISCA v2 oligonucleotide microarray platform (Oxford Gene Technology, Oxford, UK) as part of diagnostic service in the CLIA certified laboratory at The Hospital for Sick Children. Microarray experiments were performed according to the manufacturer’s instructions. Briefly, DNA from the proband and pooled same-sex reference DNA (Promega, Madison, WI, USA) were labelled with Cy3-dCTP and Cy5-dCTP, respectively, and were hybridised to the array slide. The arrays were then scanned using the Agilent G2505B microarray scanner and resulting data analysed using the CytoSure Interpret Software version 3.4.3 from Oxford Gene Technology (Begbroke, Oxfordshire, UK). CNVs on chromosome Y were removed from the analysis. Identified CNVs were classified according to ACMG^22^ guidelines and pathogenic and variant-of-unknown-significance—likely pathogenic were considered as clinically significant.

### Whole-genome sequencing

Genomic DNA was sent to Complete Genomics (Mountain View, CA, USA) for WGS as described previously.^30^ Raw sequence reads were reassembled against a reference genome (GRCh37) and variant calling was completed using Complete Genomics assembly pipeline 2.4 as previously described.^31^ This method generates a fragment of ~400 bp that is covered by a set of 8 reads totalling 70 bp sequenced. All samples passed internal Complete Genomics sample checks. Sequence results were received on hard drives and consisted of raw data plus variant calls in the form of (i) SNV and small indels, (ii) structural variants (based on abnormal junction and discordant mate-pair clusters, with size typically 50–75,000 bp), (iii) CNVs (based on normalised sequencing coverage, with size typically >2,000 bp).

### Annotation of sequence-level variants (SNVs and indels)

Complete Genomics (Mountain View, CA, USA) masterVar files were annotated using a custom pipeline based on Annovar,^32^ RefSeq gene models (downloaded from UCSC 2013 February 12), and publicly available as well as internal databases for allele frequency (1000 Genomes,^33^ NHLBI-ESP,^34^ ExAC browser (Exome Aggregation Consortium (ExAC), Cambridge, MA, USA (URL: http://exac.broadinstitute.org) January 2015), Internal Complete Genomics control databases public genomes and Wellderly population, genomic conservation (UCSC PhyloP and phastCons for placental mammals and 100 vertebrates^35^) and variant impact predictors (SIFT,^36^ PolyPhen2,^37^ Mutation Assessor,^38^ CADD^39^). We also expanded the annotation of non-coding regulatory sequence through implementation of splicing exon inclusion/exclusion predictions.^40^ Finally we annotated variants with those reported previously in disease (ClinVar,^41^ HGMD^42^) and genes reported to have abnormal mouse and human phenotype association,^29,43,44^ MGI (http://www.informatics.jax.org/). Data set and software versions are listed in the Supplementary Information.

### Diagnostic algorithm and variant interpretation

We developed a pipeline to systematically prioritise clinically significant variants (see Supplementary Figure 1). We first defined a list of high quality, rare variants (<5% population frequency) that were exonic (genes or ncRNA) or predicted to impact splicing. To aid in prioritisation variants were categorized into ordered tiers based on: (i) sequence quality; (ii) allele frequency; (iii) conservation and predicted impact on coding and non-coding sequence; and (iv) human disease and mouse abnormal phenotype association. Variants were further sorted based on: (v) their zygosity and gene mode of inheritance; and (vi) HGMD and ClinVar pathogenicity classification, reflecting implication in human genetic disorders documented in the literature (see Supplementary Information for detailed definitions). The ACMG recommendations for clinical interpretation of sequence variants^23^ were used to classify variants as pathogenic or likely pathogenic. Candidate diagnostic variants were selected based on their likely pathogenicity and explanatory power. Variants deemed diagnostic by both the assessment team and the referring physician were confirmed in the proband, and followed up in parental samples by Sanger sequencing.

### Secondary variants

The pipeline described above allowed for the identification of clinically relevant variants not related to the primary indication. The analysis was the same as for primary variants except that we focused on variants with the highest predicted impact (‘LoF’, criterion iii) and those reported to be pathogenic by HGMD or ClinVar (criterion vi). This included UTR, intronic and intergenic variants, for which impact prediction is more difficult. Finally we limited reporting of secondary variants to the 56 genes named in the 2013 ACMG guidelines on return of incidental findings.^20^

### Clinical interpretation of variants

Analysts (molecular or clinical geneticists) examined variant files and prioritized clinically relevant variants using sequence quality, allele frequency, conservation and predicted impact on coding and non-coding sequence, presence in Clinvar or HGMD, zygosity and genic mode of inheritance, and relevance to clinical phenotype provided. Candidate pathogenic variants that were interpreted to be clinically relevant to the primary or secondary phenotype were discussed with the referring clinician and designated diagnostic by consensus.

### Confirmation of sequence-level variants

Variants that were deemed to be potentially diagnostic after consultation with the clinician were confirmed with Sanger sequencing in a CLIA/CAP laboratory and a clinical report was generated. Sanger sequencing attempted for 58 variants, of which 51 of high quality (tier 2 analytic quality) had 100% confirmation rate. Parents and siblings were tested to confirm segregation for the reported pathogenic changes, or rule variants out if they did not segregate.

### WGS CNV detection and annotation

CNVs from Complete Genomics are detected through both read depth and paired-end sequencing and provided in cnvSegmentsDiploidBeta and highConfidenceSVEventsBeta files, respectively. The read depth method is based on deviation from a diploid baseline reference genome and uses 2 kb, GC-corrected windows with a hidden Markov model caller. The paired-end method by Complete Genomics employs junction detections on uniquely mapped discordant mate-pairs and can detect CNVs below 2 kb. CNVs were extracted from the cnvSegmentsDiploidBeta by parsing out segments not equal to diploid of 2 and by excluding the hypervariable regions. From the highConfidenceSVEventsBeta file we only extracted deletions and tandem duplications supported by at least 20 mate-pair reads based on previous sensitivity and specificity calculations^24^). CNVs from Chromosome Y were removed. We annotated and analysed the CNV and SV files separately. Copy number gains and losses from WGS were annotated for frequency based on 50% reciprocal overlap with CNVs called in WGS control samples, and overlap with CNVs from the Database of Genomic Variants (November 2010 and March 2013 versions);^45^ CNVs were also annotated for overlapping gene transcripts and exons (RefSeq, downloaded March 2013). Rare CNVs from either file were examined in the context of case phenotype and classified using standard criteria.^22^

### WGS comparison with CMA

The advantage of WGS over CMA for detecting CNVs is the ability to detect small CNVs, offer precise breakpoint resolution, determine the location and orientation of duplicated sequences, and find allele specific copy-number changes.^17,24^ Although WGS detects a significantly larger number of unbalanced changes compared to CMA, one of the most important questions regarding replacement of CMA with WGS in the diagnostic setting is that of accuracy. Our previous work has shown high reproducibility of CNV detection using the WGS read depth method (94%) with lower reproducibility with the paired-end method (73%).^24^ As the CNV generated from the WGS read depth method is more comparable to CMA in terms of the size detection range we determined the concordance for these methods. Previous studies and our own experience have shown that CMA lacks specificity and sensitivity in polymorphic regions overlapping with segmental duplications and affect CNV concordance between different platforms.^46^ To minimise these, we excluded CNVs showing at least 70% overlap with segmental duplications or having a frequency of least 3% in either data set. These criteria yielded 165 rare CNVs from the CMA, with 84% detected by the read depth method of WGS. Further analysis of the 26 CNVs not detected by WGS showed that the majority were in regions overlapped by segmental duplications and were labelled as ‘hypervariable’ in the WGS data. Importantly, none of these variants were identified as being clinically relevant. We examined the breakpoint concordance by comparing the difference between the CMA proximal and distal breakpoint (within probe error) to the WGS. Breakpoint analyses of the 139 concordant CNVs showed ~78% fall between the minimum and maximum boundaries of breakpoints defined by the clinical microarray, and 87% are within 10 kb of these boundaries (Supplementary Figure 9).

### WGS diagnostic yield compared to standard clinical testing

We compared the diagnostic yield of WGS to both CMA and CMA combined with targeted gene testing. Statistical differences were calculated using a *χ* ^2^ proportion test.

## Figures and Tables

**Table 1:** Molecular diagnosis rates by genetic type and mode of inheritance

	*Whole-genome sequencing*	*Clinical testing*	
		*CMA only*	*All genetic tests*
Undiagnosed	66	92	87
Diagnosis	34	8*	13**
Multiple molecular diagnosis^a^	4	0	1
			
*Type of genetic change*			
CNV only	6	8	7
SNV only	26	—	5
CNV and SNV	2	—	1
			
*Mode of Inheritance*			
Autosomal Dominant	24	8	3
Autosomal Recessive	9	0	3
Dominant and Recessive	1	0	0

Abbreviations: CNV, copy-number variants; SNV, single-nucleotide variant.

a A total of four cases had two distinct molecular diagnosis including two patients had a pathogenic CNV and SNV (see Tables 2 and 3). *χ* ^2^ proportion test *P=1.42E−05 and **P=0.0009.

**Table 2:** Clinically significant copy-number variants identified by chromosomal microarray and whole-genome sequencing

*Case ID*	*Sex*	*Clinical microarray*	*Size (Kb)*	*WGS read depth CNV*	*Origin*^a^	*Diagnosis and management*^b^
1090	F	ChrX:60,701–91,873,056 del	91,813	ChrX:176,00–91,866,000 del	DN	Partial genetic diagnosis. Category 1. Turner syndrome but hydrocephalus is not explained by molecular finding.
		ChrX:91,877,172–155,174,078 dup	63,297	ChrX:92,796,000–155,260,560 dup		
1005^c^	M	Chr4:33,574–7,608,090 del	7,575	Chr4:68,000–7,616,000 del	DN	Category 1. Wolf–Hirschhorn syndrome.
1034	F	Chr22:18,713,432–21,440,515 del	2,727	Chr22:18,888,000–21,466,000 del	DN	Category 1. 22q11.2 deletion syndrome, referred to specialized clinic.
1022	F	Chr10:30,822,400–32,872,626 del	2,050	Chr10:30,814,000–32,892,000 del	DN	Category 2. 10p11.23-p11.2 deletion. *ZEB1*; corneal dystrophy, maldevelopment of the corpus callosum.
1026	M	Chr22:35,931,002–37,272,620 del	1,342	Chr22:35,890,000–37,302,000 del	N/A	Category 3. 1.34-Mb deletion in 22q12.3.
1066	M	Chr8:97,145,564–98,301,541 del	1,156	Chr8:97,134,000–98,308,000 del	DN	Complex phenotype with two genetic disorders. Category 2. 8q22.1 *de novo* deletion. Patient also has paternally inherited *CCM2* pathogenic variant c.1054delG (p.Gly352Val*2) related to Cerebral cavernous malformation (CCM) diagnosis (Table 3).
1027	F	Chr16:15,507,164–16,400,833 del	894	Chr16:15,480,000–16,296,000 del	DN	Category 2. 16p13.11 deletion.
1102	M	Chr2:51,021,507–51,358,841del	337	Chr2:51,020,000–51,374,000 del	DN	Complex phenotype with two genetic disorders. Category 2. Pathogenic 2p16.3 microdeletion overlapping NRXN1 gene associated with global developmental delay and Autism Spectrum disorder but not able to explain episodic hypotonia and developmental regression history. Pathogenic *ATP1A3* variant c.2452G>A (p.Glu818Lys) associated with CAPOS Syndrome explains this phenotype (Table 3).

Abbreviations: DN, *de novo*; F, female; M, male; N/A, not available.

a Origin of TransmiAssion: DN; N/A.

b All findings were relevant to Genetic counselling and were further split into categories based on clinical management: Category 1 (Disease-specific published management guidelines), Category 2 (Management based on case reports or known function of genes), and Category 3 (No management change).

c In some cases the WGS CNVs were fragments and had to be manually resolved (see Supplementary Figure 8 for example in case 1005).

**Table 3:** Clinically significant sequence-level variants identified by whole-genome sequencing

*Case ID*	*Sex*	*Gene*	*IP*	*Genomic variant (zygosity)*	*Origin*^a^	*Diagnosis and management*^b^
1004	F	*EP300*	AD	c.5723dupC (p.Thr1909Asnfs*164) (het)	N/A	Category 1. Rubinstein–Taybi Syndrome 2.
1006	M	*MC4R*	AD	c.751A>C (p.Ile251Leu) (het)	MA	Partial genetic diagnosis. Category 2. Variant potentially related to obesity in proband. Mother also carries variant and has history of obesity.
1008	M	*SMARCB1*	AD	c.364del (p.Glu122Asnfs*21) (het)	N/A	Category 1. Referred for possible Noonan–Costello, found to have Coffin–Siris syndrome.
1009	M	*LARP7*	AR	c.756_757del (p.Arg253Ile*6) (hom)	MA/P	Category 2. Query RASopathy but found to have Alazami Syndrome.
1012	M	*KAT6B*	AD	c.3021+1G>C (p?) (het)	DN	Category 1. KAT6B-related disorder. Recommendation of yearly evaluations of developmental progress, contractures and/or scoliosis by an orthopaedist, ophthalmologic problems such as amblyopia (in SBBYSS), thyroid function tests, heart defects, and kidneys if hydronephrosis and/or multiple renal cysts are present.
1015	M	*GDF5*	AD	c.847G>A (p.Val283Met) (het)	DN	Category 2. Type C brachydactyly.
1016	F	*PANK2*	AR	c.824_825del (p.Cys276Trpfs*15) (hom)	MA/P	Category 2. Neurodegeneration with brain iron accumulation-1 (NBIA1).
1023	F	*NGLY1*	AR	c.1201A>T (p.Arg401*) (hom)	MA/P	Category 2. Query extrapyramidal cerebral palsy but found to have congenital disorder of deglycosylation. Initiated screening for hepatic dysfunction based on the diagnosis of NGLY1 deficiency.
1029	F	*PIK3R2*	AD	c.1117G>A (p.Gly373Arg) (het)	DN	Category 2. Megalencephaly-polymicrogyria-polydactyly-hydrocephalus syndrome-1 (MPPH). No change in management for the patient as she was outside of the age range of proposed screening recommendations for MPPH at the time of diagnosis. However, same mutation identified in her sibling who is now undergoing quarterly ultrasound surveillance for Wilms tumour. Germline mosacism suspected.
1032	F	*SPTAN1*	AD	c.6947A>C (p.Gln2316Pro) (het)	DN	Category 1. Early infantile epileptic encephalopathy 5.
1040	M	*EXT2*	AD	c.1760C>T (p.Thr587Met) (het)	N/A	Partial genetic diagnosis. Category 1. Multiple Exostoses Type 2. Radiographs confim multiple exostoses consistent with EXT variant. Need surveillance for increased cancer risk, and monitoring of exostoses growth.
1045	M	*TSEN54*	AR	c.919G>T (p.Ala307Ser) (hom)	MA/P	Category 1. Pontocerebellar Hypoplasia Type 2A.
1049	F	*NSD1*	AD	c.3922-1G>C(p?) (het)	N/A	Category 1. Query Marfan/Homocytinuria and NF type I but found to have Sotos Syndrome. Referral to appropriate specialists for management of learning disability/speech delays, behaviour problems, cardiac abnormalities, renal anomalies, scoliosis, seizures. No intervention if MRI shows ventricular dilatation without raised intracranial pressure.
1050	M	*CBL*	AD	c.1096-11_1109del (p?) (het)	DN	Category 1. Noonan Syndrome-like disorder with or without juvenile myelomonocytic leukaemia. Recommendation includes continued surveillance for leukaemia.
1055	F	*PACS1*	AD	c.607C>T (p.Arg203Trp) (het)	DN	Category 2. Autosomal dominant mental retardation 17.
1057	F	*SETD5*	AD	c.1576_1580del (p.Glu526Lysfs*15) (het)	DN	Category 2. Query Robinow syndrome but found to have Mental retardation autosomal dominant 23.
1059	M	*PIK3R1*	AD	c.1993G>A (p.Gly665Ser) (het)	P	Category 1. Query Axenfeld–Rieger syndrome but found to have SHORT syndrome. Father is also clinically affected and variant is paternally inherited. Recommended screening for diabetes and glaucoma based on the diagnosis of SHORT syndrome.
1062	M	*GJB2*	AR	c.35delG (p.Gly12fs*2) (hom)	MA/P	Partial genetic diagnosis. Category 1. Complex phenotype with autosomal recessive hearing loss caused by *GJB2* variant.
1066	M	*CCM2*	AD	c.1054delG (p.Gly352Val*2) (het)	P	Complex phenotype with two genetic disorders. Category 1. Paternally inherited *CCM2* pathogenic variant related to Cerebral cavernous malformation (CCM) diagnosis. Potential pharmacotherapy interventions for CCM. Patient also has 8q22.1 *de novo* 1.16-Mb deletion (Table 2)
1070	F	*VWF*	AD	c.6187C>T (p.Pro2063Ser) (hom)	MA/P	Partial genetic diagnosis. Category 1. Initially diagnosed with acquired Von Willebrand by haematology. However, molecular findings consistent with a genetic aetiology.
1078	M	*TYR*	AR	c.1118C>A (p.Thr373Lys) (het)/ c.1205G>A (p.Arg402Gln) (het)	MA/P	Complex phenotype with two related genetic disorders. Category 1. Oculocutaneous albinism type 1. Category 2. *MC4R* variant may be contribute to obesity
		*MC4R*	AD	c.307G>A (p.Val103Ile) (het)	N/A	
1080	M	*COL4A1*	AD	c.2317G>A (p.Gly773Arg) (het)	DN	Category 1. MRI diagnosis of *COL4A1*-related disorder confirmed with sequence testing. Recommendations for surveillance by neurologist for disease related complications including neurological, ocular, cardiac, renal and Raynaud's phenomena. Also requires aggressive hypertension management to avoid strokes.
1089	M	*PLVAP*	AR	c.1072C>T (p.Arg358*) (hom)	MA/P	Category 3. Novel protein losing enteropathy disorder.
1093	F	*NGLY1*	AR	c.517A>G (p.Arg173Gly) (hom)	MA/P	Complex phenotype with two related genetic disorders. Category 2. *NGLY1—*Congenital disorder of glycosylation, type Iv. Category 1. COG5—Congenital disorder of glycosylation, type III.
		*COG5*	AR	c.1205C>T (p.Ser402Leu) (hom)	MA/P	
1102	M	*ATP1A3*	AD	c.2452G>A (p.Glu818Lys) (het)	DN	Complex phenotype with two genetic disorders. Category 1. Pathogenic *ATP1A3* variant associated with CAPOS Syndrome explains episodic hypotonia and regression history. Patient also has pathogenic 2p16.3 337-kb deletion overlapping *NRXN1* gene associated with global developmental delay and Autism Spectrum disorder (Table 2).
1103	F	*VPS53*	AR	c.1429C>T (p.Arg477*) (het)/c.1716T>G (p.Ser572Arg) (het)	MA/P	Category 2. Pontocerebellar hypoplasia, type 2E. Older sibling with same clinical features, passed away at age 6 years, also diagnosed with the same disease afterwards. Followed in the metabolic genetics clinic for symptomatic treatment.
1107	F	*SMARCA2*	AD	c.2639 C>T (p.Thr880Ile) (het)	DN	Category 1. Nicolaides–Baraitser syndrome.
1108	F	*ALDH18A1*	AR	c.1321C>T (p.Arg441*)/ c.191G>A (p.Arg64His) (het)	MA/P	Category 2. Cutis laxa, type IIIA. Molecular diagnosis explains aetiology of similar fatal disease in sibling and enables counselling regarding recurrence risks as well as definitive prenatal diagnosis.

Abbreviations: AD, autosomal dominant; AR, autosomal recessive; DN, *de novo*; F, female; IP, inheritance pattern; M, male; MA, maternal; N/A, not available; P, paternal.

a Origin of Transmission: DN, P, MA and N/A.

b All findings were relevant to Genetic counselling and were further split into categories based on clinical management: Category 1 (Disease-specific published management guidelines); Category 2 (Management based on case reports or known function of genes); and Category 3 (No management change).

**Table 4:** Medically actionable secondary findings

*Case ID*	*Sex*	*Gene*	*IP*	*Genomic Variant (Zygosity)*	*Diagnosis*
1003	F	*FBN1*	AD	c.3509G>A p.Arg1170His (het)	Marfan syndrome
1027^a^	F	*COL3A1*	AD	c.812G>A p.Arg271Gln (het)	Ehlers-Danlos syndrome, type III; Ehlers-Danlos syndrome type IV
1040^b^	M	*SCN5A*	AD/AR	c.5239G>A p.Val1747Met (het)	Brugada syndrome 1; Cardiomyopathy, dilated, 1E; Heart block, nonprogressive; Heart block, progressive, type IA; Long QT syndrome-3; Sick sinus syndrome 1;Ventricular fibrillation, familial, 1
1063	F	*KCNH2*	AD	c.3278C>T p.Pro1093Leu (het)	Long QT syndrome; Short QT syndrome
1067	M	*SCN5A*	AD/AR	c.5336C>T p.Thr1779Met (het)	Brugada syndrome 1; Cardiomyopathy, dilated, 1E; Heart block, nonprogressive; Heart block, progressive, type IA; Long QT syndrome-3; Sick sinus syndrome 1; Ventricular fibrillation, familial, 1
1078^c^	M	*RYR2*	AD	c.3320C>T p.Thr1107Met (het)	Arrhythmogenic right ventricular dysplasia 2; Ventricular tachycardia, catecholaminergic polymorphic
1091	M	*DSG2*	AD	c.2434G>A p.Gly812Ser (het)	Arrhythmogenic right ventricular dysplasia 10; Cardiomyopathy, dilated, 1BB

Abbreviations: AD, autosomal dominant; AR, autosomal recessive; F, female; IP, inheritance pattern; M, male.

a A 894 kb (Chr16:15,507,164–16,400,833) pathogenic deletion was also detected in this patient (Table 2).

b Partial diagnosis of Multiple Exostoses Type 2 (Table 3).

c Diagnosis Oculocutaneous albinism type 1 (Table 3).
