# Supplementary material for: Whole-genome sequencing expands diagnostic utility and improves clinical management in paediatric medicine
Source: NPJ Genom Med. 2016 Jan 13;1:15012–. doi: 10.1038/npjgenmed.2015.12 (PMC5447450; doi:10.1038/npjgenmed.2015.12)
Supplement: Supplementary Information [file npjgenmed201512-s1.doc]

**Supplementary Information**

Stavropoulos *et al*. Whole Genome Sequencing Expands Diagnostic Utility and Improves Clinical Management in Pediatric Medicine

Contents

Supplementary Methods [2](#__RefHeading___Toc309585536)

Dataset and Software Versions: [2](#__RefHeading___Toc309585537)

Diagnostic pipeline category definitions [2](#__RefHeading___Toc309585538)

Supplementary Tables [4](#__RefHeading___Toc309585539)

Supplementary Table 1: Enrollment and Patient Demographics [4](#__RefHeading___Toc309585540)

Supplementary Table 2: Count of HPO terms used more than once [5](#__RefHeading___Toc309585541)

Supplementary Table 3: Counts for top level organ system level HPO terms, based on the ontology-driven up-propagation of terms used in PhenoTips [8](#__RefHeading___Toc309585542)

Supplementary Table 4: Clinical and molecular summary of patient cohort [9](#__RefHeading___Toc309585543)

Supplementary Table 5: Whole genome sequencing coverage summary [29](#__RefHeading___Toc309585544)

Supplementary Table 6: Whole genome sequencing variant summary [31](#__RefHeading___Toc309585545)

Supplementary Table 7: Whole genome sequencing CNV and SV summary [32](#__RefHeading___Toc309585546)

Supplementary Table 8: Illustrative case examples and impact on clinical management [33](#__RefHeading___Toc309585547)

Supplementary Table 9: Clinically relevant exonic deletions [34](#__RefHeading___Toc309585548)

Supplementary Figures [35](#__RefHeading___Toc309585549)

Supplementary Figure 1: Overview of WGS Analysis [35](#__RefHeading___Toc309585550)

Supplementary Figure 2: Histogram of frequency of HPO terms used in the cohort [36](#__RefHeading___Toc309585551)

Supplementary Figure 3: Histogram of number of HPO terms used to describe phenotypes [37](#__RefHeading___Toc309585552)

Supplementary Figure 4: Stacked histogram of relative diagnostic rate across the common phenotypes in the cohort [38](#__RefHeading___Toc309585553)

Supplementary Figure 5: Stacked histogram of relative diagnostic rate across major HPO terms [39](#__RefHeading___Toc309585554)

Supplementary Figure 6: Deletion at intron-exon boundary in *CBS* gene [40](#__RefHeading___Toc309585555)

Supplementary Figure 7: Binned Copy Number count in cohort using different detection methods [41](#__RefHeading___Toc309585556)

Supplementary Figure 8: *De novo* 7.6 Mb deletion at 4p16.3-p16.1 [42](#__RefHeading___Toc309585557)

Supplementary Figure 9: Beakpoint concordance of CNVs called in WGS and CMA [43](#__RefHeading___Toc309585558)

Supplementary References: [44](#__RefHeading___Toc309585559)

# Supplementary Methods

## Dataset and Software Versions:

- All databases are referred to hg19 genome build.
- Annovar: (Nov 2014 version)
- Annovar database for 1000G: 2014 Oct version.
- Annovar database for NHLBI-ESP: esp6500si version (downloaded Jun 2012)
- Annovar database for Exac database (downloaded Nov 2014)
- Annovar databases for SIFT, PolyPhen2 HVAR, MutationAssessor, MutationTaster: ljb26 (downloaded Sept 2014 ), based on dbNSFP.
- Annovar database for CADD (downloaded March 2014)
- dbSNP: version 138.
- Clinvar (downloaded Sept 2014).
- Cosmic: version 70.
- HGMD: licensed commercial version, downloaded Aug 2014.
- RefSeq: RefGene table, downloaded from UCSC Jan 2015.
- OMIM: morbidmap downloaded Jan 2015.
- CGD, HPO, MGI/MPO: downloaded and processed Sept/Nov 2014.
- PhastCons placental mammal: downloaded from UCSC Dec 2009.
- SegDups: downloaded from UCSC Oct 2011.
- PhyloP placental mammals: downloaded from UCSC Nov 2009
- PhyloP 100 vertebrates: downloaded from UCSC Dec 2014.
- Repeats: downloaded from UCSC Jun 2013.
- PFAM: downloaded from UCSC Nov 2014.

## Diagnostic pipeline category definitions

*(i) Sequence quality***.**

Quality tier 1 was defined as those passing Complete Genomics default quality filter (VarQuality = PASS), excluding no-calls and half-calls (i.e. where only one allele could be called with sufficient confidence). Quality tier 2 additionally required minimum depth of 5 reads, estimated ploidy of 2 or 1 (where ploidy 1 additionally required a homozygous call, suggesting a hemizygous deletion, or haploid zygosity, assigned only to male non-pseudoautosomal X regions), higher variant quality scores (VarScoreEAF > 40 for heterozygous and > 20 for homozygous calls, no additional requirements for haploid calls), alternate allele support compatible with zygosity (>= 0.30 for heterozygous calls and >= 0.80 for homozygous calls). While variants overlapping segmental duplications in principle could achieve quality tier 2, most of them have undetermined estimated ploidy, and thus fail the ploidy requirement for quality tier-2; in any case, quality tier-2 variants overlapping segmental duplications where considered more skeptically.

(ii) *Allele frequency.*

All variants were categorized into allele frequency tiers (<= 5%, <= 1%, <= 0.5%, novel) based on the maximum allele frequency from 1000 Genomes, 54 unrelated Complete Genomics genomes from the multi-ethnic reference panel, NHLBI-ESP, ExAC, the Wellderly Complete Genomics control population (597 Caucasian subjects), the Complete Genomics 1000 genome subset (436 subjects).

(iii) *Conservation and predicted impact on gene product***.**

Different definitions were used for protein coding versus ncRNA and no attempt was made at predicting UTR or intergenic sequence as damaging; for intronic sequence, only the predicted effect on splicing was considered. Frameshift insertions/deletions/substitutions, substitutions creating a stop codon gain and alterations of the intronic dinucleotide adjacent to a coding-exonic splice junction were classified as “LoF” (loss of function) and were assigned to damaging tier 2. Damaging tier 2 was assigned to missense variants passing set thresholds for at least 4/7 impact predictors and conservation scores (SIFT < 0.05, PolyPhen2 HVAR >= 0.90, MutationAssessor >= 1.90, PhyloP placental mammals >= 2.30, PhyloP 100 vertebrates >= 4.00, Phred-scaled CADD >= 15, MutationTaster >= 0.5); damaging tier 1 was assigned to missense variants passing set thresholds for less than 4 / 7 but at least 2 / 7 impact predictors and conservation scores. Coding variants consisting of multi-nucleotide substitutions or insertions/deletions not causing frameshift, or any type of variant causing loss of the stop codon, were assigned to damaging tier 1 if (a) did not overlap a dbSNP common variant and had Phred-scaled CADD >= 20 or PhyloP placental mammals >= 2.30 or PyloP 100 vertebrates >= 4.00, or (b) did not overlap a dbSNP variant and had Phred-scaled CADD >= 15 or PhyloP placental mammals >= 1.50 or PyloP 100 vertebrates >= 2.50. Damaging tier 2 was assigned to splicing-regulatory variants with change in percentage splicing inclusion (dPSI) <= -5. Damaging tier 1 was assigned to splicing-regulatory variants with -2.5 >= dPSI > -5 or dPSI >= 5. Non-coding gene exonic or core splice-site variants were assigned to damaging tier 2 if they overlapped a mammalian PhastCons conserved element and had Phred-scaled CADD >= 17.5 or PhyloP placental mammals >= 2.50 or PhyloP 100 vertebrates >= 4.50. They were assigned to damaging tier 1 if they did not match criteria for tier-1 but had Phred-scaled CADD >= 15 or if they overlapped a mammalian PhastCons conserved element and had PhyloP placental mammals >= 2.00 or PhyloP 100 vertebrates >= 3.50.

(iv) *Human disease and mouse abnormal phenotype.* The phenotype tier captures the likelihood of the gene to produce the desired phenotype when perturbed. Tier 1 captures any gene with an associated phenotype in human or mouse and Tier 2 captures genes with a phenotype compatible with a pre-composed selection based on cases specific HPO terms exported from phenotips information.

(v) *Zygosity and gene mode of inheritance***.** To prioritize variants predictive of disease state we further parsed the list of rare variants into groups of variants based on zygosity and the mode of inheritance reported for the gene (see Supplemental Figure 1). For the primary pipeline we used these definitions:

*Autosomal Dominant* group (AD): Variants <= 0.5% frequency impacting genes with dominant mode of inheritance as defined only for genes with a disease or abnormal phenotype in humans (HPO, CGD).

*Homozygotes* group (AR-Hom):Variants at <= 5% frequency that are homozygous, regardless of the genes implication in disease/abnormal phenotype and relative mode of inheritance.

*Potential Compound Heterozygotes* group (AR-CH): Sets of two or more variants per gene at <= 5% frequency. In absence of parents or short-range read-backed phasing, only “potential” compound heterozygotes can be identified.

*Male X-linked* group (XL):

X chromosome variants that are haploid (thus only in non-pseudoautosomal regions of males), regardless of the genes implication in disease/abnormal phenotype and relative mode of inheritance.

# Supplementary Tables

## Supplementary Table 1: Enrollment and Patient Demographics

| **Families Contacted (n=201)** |  |
| --- | --- |
| **Enrolled** | 100 |
| **Declined** | 95 |
| **Undecided** | 6 |
| **Mean time to consent** | 10.8 days |
| **Enrolled Demographics (n=100)** |  |
| **Mean Age** | 5yr 5months |
| **Age Range** | <1month-18years |
| **<5 years of Age** | 58% |
| **< 1year of Age** | 25% |
| **Female:Male** | 43:57 |
| **Reported Consanguinity** | 8% |
| **Secondary Findings for Adult onset disorders (n=100)** |  |
| **Yes:No:Undecided** | 67:26:7 |

## Supplementary Table 2: Count of HPO terms used more than once

| **HPO.ID** | **HPO.Name** | **PhenoTipsCount** |
| --- | --- | --- |
| HP:0001263 | Global developmental delay | 29 |
| HP:0004325 | Decreased body weight | 24 |
| HP:0002194 | Delayed gross motor development | 23 |
| HP:0000252 | Microcephaly | 22 |
| HP:0004322 | Short stature | 20 |
| HP:0001250 | Seizures | 18 |
| HP:0000750 | Delayed speech and language development | 17 |
| HP:0001290 | Generalized hypotonia | 15 |
| HP:0010862 | Delayed fine motor development | 13 |
| HP:0001999 | Abnormal facial shape | 10 |
| HP:0004209 | Clinodactyly of the 5th finger | 8 |
| HP:0000218 | High palate | 7 |
| HP:0000431 | Wide nasal bridge | 7 |
| HP:0001629 | Ventricular septal defect | 7 |
| HP:0002020 | Gastroesophageal reflux | 7 |
| HP:0010864 | Intellectual disability, severe | 7 |
| HP:0000256 | Macrocephaly | 6 |
| HP:0000278 | Retrognathia | 6 |
| HP:0000347 | Micrognathia | 6 |
| HP:0000463 | Anteverted nares | 6 |
| HP:0001332 | Dystonia | 6 |
| HP:0001643 | Patent ductus arteriosus | 6 |
| HP:0004324 | Increased body weight | 6 |
| HP:0004691 | 2-3 toe syndactyly | 6 |
| HP:0005280 | Depressed nasal bridge | 6 |
| HP:0000316 | Hypertelorism | 5 |
| HP:0000369 | Low-set ears | 5 |
| HP:0000664 | Synophrys | 5 |
| HP:0001328 | Specific learning disability | 5 |
| HP:0001631 | Defect in the atrial septum | 5 |
| HP:0001655 | Patent foramen ovale | 5 |
| HP:0011398 | Central hypotonia | 5 |
| HP:0000047 | Hypospadias | 4 |
| HP:0000325 | Triangular face | 4 |
| HP:0000403 | Recurrent otitis media | 4 |
| HP:0000505 | Visual impairment | 4 |
| HP:0000708 | Behavioral abnormality | 4 |
| HP:0000729 | Autistic behavior | 4 |
| HP:0000953 | Hyperpigmentation of the skin | 4 |
| HP:0000954 | Single transverse palmar crease | 4 |
| HP:0001256 | Intellectual disability, mild | 4 |
| HP:0002079 | Hypoplasia of the corpus callosum | 4 |
| HP:0002376 | Developmental regression | 4 |
| HP:0009891 | Underdeveloped supraorbital ridges | 4 |
| HP:0000023 | Inguinal hernia | 3 |
| HP:0000219 | Thin upper lip vermilion | 3 |
| HP:0000238 | Hydrocephalus | 3 |
| HP:0000286 | Epicanthus | 3 |
| HP:0000324 | Facial asymmetry | 3 |
| HP:0000341 | Narrow forehead | 3 |
| HP:0000365 | Hearing impairment | 3 |
| HP:0000414 | Bulbous nose | 3 |
| HP:0000455 | Broad nasal tip | 3 |
| HP:0000490 | Deeply set eye | 3 |
| HP:0000565 | Esotropia | 3 |
| HP:0000582 | Upslanted palpebral fissure | 3 |
| HP:0000717 | Autism | 3 |
| HP:0000767 | Pectus excavatum | 3 |
| HP:0000960 | Sacral dimple | 3 |
| HP:0000964 | Eczema | 3 |
| HP:0001028 | Hemangioma | 3 |
| HP:0001257 | Spasticity | 3 |
| HP:0001382 | Joint hypermobility | 3 |
| HP:0001508 | Failure to thrive | 3 |
| HP:0001513 | Obesity | 3 |
| HP:0001537 | Umbilical hernia | 3 |
| HP:0001680 | Coarctation of aorta | 3 |
| HP:0002015 | Dysphagia | 3 |
| HP:0002342 | Intellectual disability, moderate | 3 |
| HP:0002650 | Scoliosis | 3 |
| HP:0004467 | Preauricular pit | 3 |
| HP:0008070 | Sparse hair | 3 |
| HP:0009765 | Low hanging columella | 3 |
| HP:0011471 | Gastrostomy tube feeding in infancy | 3 |
| HP:0000028 | Cryptorchidism | 2 |
| HP:0000098 | Tall stature | 2 |
| HP:0000248 | Brachycephaly | 2 |
| HP:0000280 | Coarse facial features | 2 |
| HP:0000308 | Microretrognathia | 2 |
| HP:0000356 | Abnormality of the outer ear | 2 |
| HP:0000358 | Posteriorly rotated ears | 2 |
| HP:0000377 | Abnormality of the pinna | 2 |
| HP:0000426 | Prominent nasal bridge | 2 |
| HP:0000430 | Underdeveloped nasal alae | 2 |
| HP:0000470 | Short neck | 2 |
| HP:0000494 | Downslanted palpebral fissures | 2 |
| HP:0000520 | Proptosis | 2 |
| HP:0000525 | Abnormality of the iris | 2 |
| HP:0000574 | Thick eyebrow | 2 |
| HP:0000592 | Blue sclerae | 2 |
| HP:0000612 | Iris coloboma | 2 |
| HP:0000668 | Hypodontia | 2 |
| HP:0000678 | Dental crowding | 2 |
| HP:0001010 | Hypopigmentation of the skin | 2 |
| HP:0001027 | Soft, doughy skin | 2 |
| HP:0001182 | Tapered finger | 2 |
| HP:0001276 | Hypertonia | 2 |
| HP:0001298 | Encephalopathy | 2 |
| HP:0001371 | Flexion contracture | 2 |
| HP:0001385 | Hip dysplasia | 2 |
| HP:0001511 | Intrauterine growth retardation | 2 |
| HP:0001539 | Omphalocele | 2 |
| HP:0001601 | Laryngomalacia | 2 |
| HP:0001647 | Bicuspid aortic valve | 2 |
| HP:0001763 | Pes planus | 2 |
| HP:0001773 | Short foot | 2 |
| HP:0001814 | Deep-set nails | 2 |
| HP:0001838 | Rocker bottom foot | 2 |
| HP:0001864 | Clinodactyly of the 5th toe | 2 |
| HP:0002007 | Frontal bossing | 2 |
| HP:0002011 | Morphological abnormality of the central nervous system | 2 |
| HP:0002088 | Abnormality of the lung | 2 |
| HP:0002092 | Pulmonary hypertension | 2 |
| HP:0002307 | Drooling | 2 |
| HP:0002373 | Febrile seizures | 2 |
| HP:0002453 | Abnormality of the globus pallidus | 2 |
| HP:0002575 | Tracheoesophageal fistula | 2 |
| HP:0002652 | Skeletal dysplasia | 2 |
| HP:0002705 | High, narrow palate | 2 |
| HP:0003072 | Hypercalcemia | 2 |
| HP:0004442 | Sagittal craniosynostosis | 2 |
| HP:0005750 | Contractures of the joints of the lower limbs | 2 |
| HP:0005815 | Supernumerary ribs | 2 |
| HP:0006532 | Recurrent pneumonia | 2 |
| HP:0007099 | Arnold-Chiari type I malformation | 2 |
| HP:0007633 | Bilateral microphthalmos | 2 |
| HP:0007930 | Prominent epicanthal folds | 2 |
| HP:0008404 | Nail dystrophy | 2 |
| HP:0008947 | Infantile muscular hypotonia | 2 |
| HP:0009473 | Joint contracture of the hand | 2 |
| HP:0009889 | Localized hirsutism | 2 |
| HP:0011968 | Feeding difficulties | 2 |
| HP:0012444 | Brain atrophy | 2 |
| HP:0100490 | Camptodactyly of finger | 2 |
| HP:0100876 | Infra-orbital crease | 2 |
| HP:0200007 | Abnormal size of the palpebral fissures | 2 |
| HP:0200055 | Small hand | 2 |

## Supplementary Table 3: Counts for top level organ system level HPO terms, based on the ontology-driven up-propagation of terms used in PhenoTips

| **HPO.ID** | **HPO.name** | **Case Count** |
| --- | --- | --- |
| HP:0000707 | Abnormality of the nervous system | 77 |
| HP:0000152 | Abnormality of head and neck | 70 |
| HP:0000924 | Abnormality of the skeletal system | 68 |
| HP:0001507 | Growth abnormality | 44 |
| HP:0001574 | Abnormality of the integument | 38 |
| HP:0000478 | Abnormality of the eye | 34 |
| HP:0001626 | Abnormality of the cardiovascular system | 32 |
| HP:0003011 | Abnormality of the musculature | 27 |
| HP:0000598 | Abnormality of the ear | 26 |
| HP:0001438 | Abnormality of the abdomen | 25 |
| HP:0000119 | Abnormality of the genitourinary system | 18 |
| HP:0002086 | Abnormality of the respiratory system | 16 |
| HP:0003549 | Abnormality of connective tissue | 13 |
| HP:0002715 | Abnormality of the immune system | 9 |
| HP:0001939 | Abnormality of metabolism/homeostasis | 7 |
| HP:0001871 | Abnormality of blood and blood-forming tissues | 6 |
| HP:0002664 | Neoplasm | 5 |
| HP:0000818 | Abnormality of the endocrine system | 5 |
| HP:0000769 | Abnormality of the breast | 2 |
| HP:0001197 | Abnormality of prenatal development or birth | 1 |
| HP:0001608 | Abnormality of the voice | 1 |

## Supplementary Table 4: Clinical and molecular summary of patient cohort

| **Case ID** | **Sex^1^** | **Clinical Indication and Phenotype (Summary)** | **Genetic tests ordered** | **#Genetic Tests** | **Standard Testing Dx** | **WGS Dx** | **Gene (NM) or Locus** | **IP^2^** | **Genomic Variant (zygosity)** | **Origin^3^** | **Diagnosis and Management (4)** |
| --- | --- | --- | --- | --- | --- | --- | --- | --- | --- | --- | --- |
| 1000 | M | Global Developmental Delay, ID, Seizures, Hypotonia | Microarray, Karyotype | 2 | No | No | - | - | - | - | No specific clinical or molecular diagnosis |
| 1001 | M | Rhizomelic leg/arm shortening, fibular hyoplasia, postaxial oligodactyly | Microarray | 1 | No | No | - | - | - | - | No specific clinical or molecular diagnosis, presentation suggests patterning defect |
| 1002 | M | Microcephaly and MCA, seizures and failure to thrive, profound mental retardation | Microarray, FISH 22q11.2, X-Linked MR Panel (Ambry Genetics), Karyotype, Subtelomeric FISH | 5 | No | No | - | - | - | - | No specific clinical or molecular diagnosis |
| 1003 | F | Seizures, strokes, possible connective tissue disease | Microarray | 1 | No | No | - | - | - | - | No specific clinical or molecular diagnosis, potential vasculopathy |
| 1004 | F | Lipomyelomeningocele, microcephaly, developmental delay | Microarray | 1 | No | Yes | EP300 (NM_001429.3) | AD | c.5723dupC (p.Thr1909Asnfs*164) (het) | N/A | Category 1. Rubinstein-Taybi Syndrome 2 |
| 1005 | M | Hypotonia and multiple congenital aNomalies | Microarray | 1 | Yes | Yes | 4p16.3-p16.1 | AD | arr 4p16.3p16.1(72,320-7,608,090)x1 | DN | Category 1. Wolf-Hirschhorn Syndrome. |
| 1006 | M | Mild Global Developmental Delay, chronic diarhea, disglycemia, obesity | Microarray | 1 | No | Partial | MC4R (NM_005912) | AD | c.751A>C (p.Ile251Leu) (het) | M | Partial genetic diagnosis. Category 2. Variant potentially related to obesity in proband. Mother also carries variant and has history of obesity. |
| 1007 | F | Global Developmental Delay; generalized hypothonia | Microarray, PMP22 Dosage, SMN1 Dosage | 3 | No | No | - | - | - | - | No specific clinical or molecular diagnosis. Possible mitochondrial/metabolic condition |
| 1008 | M | Global Developmental Delay, Scoliosis, joint hypermobility, hypoplasia of the corpus callosum, facial dysmorphology | Microarray, Noonan Panel (Harvard), Fragile X, 22q11.2 Dosage | 4 | No | Yes | SMARCB1 (NM_003073.3) | AD | c.364del (p.Glu122Asnfs*21) (het) | N/A | Category 1. Referred for possible Noonan-Costello, found to have Coffin-Siris syndrome. |
| 1009 | M | Developmental Delay, Short Stature, Metaphyseal Dysplasia | Microarray, Noonan Panel (Harvard), SNRPN for Prader-Willi, Fragile X, 22q11.2 Dosage, 15q11.2 Dosage | 6 | No | Yes | LARP7 (NM_016648.2) | AR | c.756_757del (p.Arg253Ile*6) (hom) | M/P | Category 2. Query RASopathy but found to have Alazami Syndrome. |
| 1010 | F | Intractable infantile onset myoclonic epilepsy, developmental delay, esotropia, generalized hypotonia | Microarray, Clinical Exome (Baylor), Comprehensive Epilepsy Panel (GeneDx), SickKids NCL Panel | 5 | No | No | - | - | - | - | No specific clinical or molecular diagnosis |
| 1011 | F | Global Developmental Delay, ID, Cardiomyopathy | Microarray, Karyotype, Recessive EDS Panel (CTGT), Sequencing of FBN1, TGFBR1, TGFBR2, ACTA1, NEB, SEPN1, COL6A1, COL6A2, COL6A3, and SMAD3 | 13 | No | No | - | - | - | - | Connective tissue disorder, query Marfan/Loeys-Dietz syndrome. No molecular diagnosis |
| 1012 | M | Neonatal encephalopathy | Microarray | 1 | No | Yes | KAT6B (NM_012330.3) | AD | c.3021+1G>C (p?) (het) | DN | Category 1. KAT6B-Related Disorder. Recommendation of yearly evaluations of developmental progress, contractures and/or scoliosis by an orthopedist, ophthalmologic problems such as amblyopia (in SBBYSS), thyroid function tests, heart defects, and kidneys if hydronephrosis and/or multiple renal cysts are present. |
| 1013 | M | Glossoptosis, Micrognathia, Arnold-Chiari type I malformation | Microarray, Fragile X | 2 | No | No | - | - | - | - | Pierre-Robin sequence and hypodontia. No molecular diagnosis |
| 1014 | F | Global developmental delay, Ventricular septal defect | Microarray, Karyotype, FISH 22q11.2, FISH 7q11.23, Comprehensive Mitochondrial Nuclear Gene Panel Seqencing and Dosage, | 6 | No | No | - | - | - | - | No specific clinical or molecular diagnosis |
| 1015 | M | Brachydactyly of the second digit, ulnar deviation of the fingers, single transverse palmar crease bilaterally, left talipes equinovarus | Microarray | 1 | No | Yes | GDF5 (NM_000557.2) | AD | c.847G>A (p.Val283Met) (het) | DN | Category 2. Type C Brachydactyly |
| 1016 | F | Global Developmental Delay | Microarray | 1 | No | Yes | PANK2 (NM_153638.2) | AR | c.824_825del (p.Cys276Trpfs*15) (hom) | M/P | Category 2. Neurodegeneration with brain iron accumulation-1 (NBIA1). |
| 1018 | F | Undergrowth with Normal head circumference | Microarray, Karyotype, Methylation of chr11, 11p15.5 Dosage, UPD 7 Testing | 5 | No | No | - | - | - | - | Query Russell-Silver Syndrome. No molecular diagnosis |
| 1019 | M | Fetal akinesia, microcephaly, contractures, myasthenia | Microarray, Fetal Akinesia Panel (Prevention Genetics) Sequence and Dosage | 3 | No | No | - | - | - | - | No specific clinical or molecular diagnosis |
| 1020 | M | Multiple congenital anomalies | Microarray | 1 | No | No | - | - | - | - | No specific clinical or molecular diagnosis |
| 1021 | M | Congenital hydrocephalus with ventriculomegaly | Microarray | 1 | No | No | - | - | - | - | No specific clinical or molecular diagnosis |
| 1022 | F | Mild global developmental delay, Autistic behavior, agenesis of corpus callosum, keratoconus | Microarray | 1 | Yes | Yes | 10p11.23-p11.22 | AD | arr 10p11.23p11.22(30,822,400-32,872,150)x1 | DN | Category 2. 10p11.23-p11.2 deletion. ZEB1; corneal dystrophy, maldevelopment of the corpus callosum. |
| 1023 | F | Global developmental delay, Non-ambulatory since age 4, absent speech, distal muscular atrophy, choreoathetosis, possible seizures, keratoconus | Microarray, Karyotype, Fragile X, Angelman methylation, MECP2 Dosage, Sequencing of STK9, UBE3A, and CDKL5 | 8 | No | Yes | NGLY1 (NM_018297.3) | AR | c.1201A>T (p.Arg401*) (hom) | M/P | Category 2. Query extrapyramidal cerebral palsy but found to have congenital disorder of deglycosylation. Initiated screening for hepatic dysfunction based on the diagnosis of NGLY1 deficiency. |
| 1024 | F | Multiple congenital anomalies, patent ductus arteriosus, hip dysplasia | Microarray | 1 | No | No | - | - | - | - | No specific clinical or molecular diagnosis |
| 1025 | F | Global Developmental Delay, microcephaly, dysmorphic features | Microarray, Sequencing of PTPN11, SOS1, KRAS, RAF1 | 5 | No | No | - | - | - | - | Query RASopathy. No molecular diagnosis |
| 1026 | M | Global Developmental Delay, congential heart defect | Microarray, 22q12.2 FISH | 2 | Yes | Yes | 22q12.2 | AD | arr 22q12.2 (Chr22: 35,931,002-37,272,620)x1 | N/A | Category 3. Likely pathogenic 1.34 Mb deletion. |
| 1027 | F | Chorioretinal lacunae and hypoplastic corpus callosum, intractable epilepsy | Microarray, Sequencing of NDE1 | 2 | Yes | Yes | 16p13.11 | AD | arr 16p13.11(15,507,164-16,400,833)x1 | DN | Category 2. 16p13.11 deletion. |
| 1028 | M | Extreme prematurity, global developmental delay, rhabdomyolysis, dysmorphic features | Microarray, Sequencing and Dosage of LPIN1 and RYR1, rhabdomyolysis panel | 5 | No | No | - | - | - | - | Childhood recurrent acute myoglobinuria. No molecular diagnosis |
| 1029 | F | Macrocephaly, polymicrogyria, somatic asymmetry,echogenic kidneys, spondylolisthesis, mild developmental delay (sibling with similar phenotype: macrocephaly, polymicrogyria, Chiari malformation, speech delay) | Microarray, Sequencing of PTEN | 2 | No | Yes | PIK3R2 (NM_181523.2) | AD | c.1117G>A (p.Gly373Arg) (het) | DN | Category 2. Megalencephaly-polymicrogyria-polydactyly-hydrocephalus syndrome-1 (MPPH). No change in management for the patient as she was outside of the age range of proposed screening recommendations for MPPH at the time of diagnosis. However, same mutation identified in her sibling who is now undergoing quarterly ultrasound surveillance for Wilms tumor. Germline mosacism suspected. |
| 1030 | M | Dysmorphic features and critical aortic stenosis | Microarray, Karyotype, 22q11.2 FISH, Sequencing and Dosage of TGFBR1 and TGFBR2 | 7 | No | No | - | - | - | - | Query Connective tissue disease. No molecular diagnosis |
| 1031 | F | Short stature, developmental delay, pulmonary artery stenosis, seizures and vision problems | Microarray, Noonan Panel (GeneDx), Sanger Sequencing of NF1, Methylation studies for RSS | 4 | No | No | - | - | - | - | Query Noonan syndrome or Methylation defect. Presentation is non-specific developmental delay and short stature. No molecular diagnosis |
| 1032 | F | Microcephaly, hypotonia and intractable seizures | Microarray, Sequencing of SPTAN1 | 2 | Yes | Yes | SPTAN1 (NM_001130438.2) | AD | c.6947A>C (p.Gln2316Pro) (het) | DN | Category 1. Early Infantile Epileptic Encephalopathy 5. |
| 1034 | F | Multiple congenital anomalies; query 22q11.2DS | Microarray, 22q12.2 del FISH | 2 | Yes | Yes | 22q11.21 | AD | arr 22q11.21(18,713,432-21,440,515)x1 | DN | Category 1. 22q11.2 Deletion syndrome, referred to specialized clinic. |
| 1035 | M | Constitutional overgrowth, autistic spectrum disorder, ADHD, pectus excavatum, advanced boneage | Microarray, Fragile X, Seqencing and dosage for PTEN, FMR1, GPC3 | 5 | No | No | - | - | - | - | Unspecificed overgrowth syndrome. No specific clinical or molecular diagnosis |
| 1036 | M | Autism spectrum disorder, bilateral inguinal hernias, positive family history of Beckwith-Wiedemann syndrome | Microarray | 1 | No | No | - | - | - | - | Autism spectrum disorder. No molecular diagnosis. Idential twin with molecularly confirmed BWS |
| 1038 | M | Leg length discrepancy and deformity with multiple enchondromas | Microarray | 1 | No | No | - | - | - | - | Query Ollier's disease. No molecular diagnosis |
| 1039 | M | Pierre-Robin sequence | Microarray | 1 | No | No | - | - | - |  | Pierre-Robin sequence. No molecular diagnosis |
| 1040 | M | Hypophosphatemic Rickets | Microarray, Sequencing and dosage PHEX, FGF23, DMP1 and Sequencing ENPP1 | 6 | No | Partial | EXT2 (NM_207122.1) | AD | c.1760C>T (p.Thr587Met) (het) | N/A | Partial genetic diagnosis. Category 1. Multiple Exostoses Type 2. Radiographs confim multiple exostoses consistent with EXT2 variant. Need surveillance for increased cancer risk, and monitoring of exostoses growth. |
| 1041 | F | Global Developmental delay, Congenital anomalies, Dysmorphic features, Atism | Microarray, Coffin-Siris Panel Sequencing and Dosage, Sequencing and Dosage of SMARCB1, MECP2, and Sequencing of CHD7 | 8 | No | No | - | - | - | - | Query CHARGE or Coffin-Siris. No molecular diagnosis |
| 1042 | M | "Cutis Marmorata, Developmental delay, Seizures, Microcephaly, Dysmorphic Features, | Microarray, 17p.11.2 FISH, karyotype, Seqencing and Dosage NDP, CDKL5, MECP2 | 7 | No | No | - | - | - | - | No specific clinical or molecular diagnosis |
| 1043 | M | VACTERL association, single kidney, anorectal malformation, vertebral abnormalities, | Microarray | 1 | No | No | - | - | - | - | Query VACTERL. No molecular diagnosis |
| 1044 | M | Cleft palate, seizures | Microarray, 22q11.2 FISH | 2 | No | No | - | - | - | - | No specific clinical or molecular diagnosis |
| 1045 | M | Choreoatethosis, Developmental delay, Pontocerebellar Hypoplasia, Feeding problems | Microarray, Sequencing TSEN54 | 2 | Yes | Yes | TSEN54 (NM_207346.2) | AR | c.919G>T (p.Ala307Ser) (hom) | M/P | Category 1. Pontocerebellar Hypoplasia Type 2A. |
| 1046 | M | Global developmental delay, perinatal hypoxic-ischemic encephalopathy, acquired microcephaly, dysmorphic features | Microarray, Sequencing and Dosage GLI3 | 3 | No | No | - | - | - | - | No specific clinical or molecular diagnosis |
| 1047 | F | Developmental delay, learning disabilities, developmental regression, querry Asperger's syndrome, idiopathic urticard and angioedema, generalized body pain and fevers NYD. | Microarray | 1 | Yes | No | - | - | - | - | No specific clinical or molecular diagnosis |
| 1048 | M | Febrile seizures, recurrent facial swelling, speech delay | Microarray, Periodic Fever Syndromes Panel (GeneDx) | 2 | No | No | - | - | - | - | Developmental Delay and Autoimmune Disorder. No molecular diagnosis |
| 1049 | F | Marfanoid habitus, mild developmental delay late onset, delayed gross motor development. | Microarray, Sequencing and Dosage FBN1 | 3 | No | Yes | NSD1 (NM_022455.4) | AD | c.3922-1G>C (p.?) (het) | N/A | Category 1. Query Marfan/Homocytinuria and NF type I but found to have Sotos Syndrome. Referral to appropriate specialists for management of learning disability/speech delays, behavior problems, cardiac abnormalities, renal anomalies, scoliosis, seizures. No intervention if MRI shows ventricular dilatation without raised intracranial pressure. |
| 1050 | M | Antenatal diagnosis of JMML(Juvenile Myelomonocytic Leukemia) and pulmonary stenosis and dysmorphism | Microarray, Noonan panel (Harvard), chromosome breakage | 3 | No | Yes | CBL (NM_005188.3) | AD | c.1096-11_1109del (p.?) (het) | DN | Category 1. Noonan Syndrome-like disorder with or without juvenile myelomonocytic leukemia. Recommendation includes continued surveillance for leukemia. |
| 1051 | F | Recurrent hypoglycemia (combined postprandial and prolonged fasting); prematurity related spastic diplegia; focal dystonia | Microarray, Sequencing and Dosage GYS1, GYS2, GLUD1, dystonia sequencing panel | 8 | No | No | - | - | - | - | No specific clinical or molecular diagnosis |
| 1052 | M | Hypotonia and developmental delay with regression of milestones, recurrent pulmonary infections requiring multiple intubations, phenocopy of Niemann-Pick syndrome | Microarray + GATM seq + NPC1 seq+ SNRPN MLPA Prader-Willi | 4 | No | No | - | - | - | - | No specific clinical or molecular diagnosis |
| 1053 | F | Omphalocele, PDA | Microarray | 1 | No | No | - | - | - | - | No specific clinical or molecular diagnosis |
| 1055 | F | Failure to thrive, seizures, macrocephaly with communicating hydrocephalus, increased axial CSF, GI reflux, myopia, hypotonia, borderline QT,astigmatic, bicuspid aortic valve, cyclic neutropenia | Microarray, epilepsy panel (Courtagen) | 2 | No | Yes | PACS1 (NM_018026.3) | AD | c.607C>T (p.Arg203Trp) (het) | DN | Category 2. Autosomal Dominant Mental Retardation 17. |
| 1056 | M | laryngomalacia and feeding difficulties | Microarray | 1 | No | No | - | - | - | - | No specific clinical or molecular diagnosis |
| 1057 | F | Hypotonia and developmental delay and Non-specific skeletal abnormalities. | Microarray, Sequencing and Dosage of WNT5A and ROR2, Sequencing FGFR3 | 5 | No | Yes | SETD5 (NM_001080517) | AD | c.1576_1580del (p.Glu526Lysfs*15) (het) | DN | Category 2. Query Robinow syndrome but found to have Mental retardation autosomal dominant 23. |
| 1058 | M | Hypotonia, sacral dimple, syndactyly | Microarray | 1 | No | No | - | - | - | - | No specific clinical or molecular diagnosis |
| 1059 | M | Axenfeld-Rieger anomaly, hypodontia, distinctive facial features | Microarray, Sequencing and Dosage PITX2 and FOXC1 | 4 | No | Yes | PIK3R1 (NM_181523.2) | AD | c.1993G>A (p.Gly665Ser) (het) | P | Category 1. Query Axenfeld-Rieger syndrome but found to have SHORT syndrome. Father is also clinically affected and variant is paternally inherited. Recommended screening for diabetes and glaucoma based on the diagnosis of SHORT syndrome. |
| 1060 | M | Developmental delay, hypotonia, pervasive developmental disorder | Microarray, Fragile X | 2 | No | No | - | - | - | - | No specific clinical or molecular diagnosis |
| 1061 | F | Hand and foot anomalies (Amputations of digits and duplications) | Microarray, Sequencing and Dosage ARHGAP31, DOCK6, EOGT, and RBPJ | 6 | No | No | - | - | - | - | Query Adams-Oliver syndrome. No molecular diagnosis |
| 1062 | M | Sensorineural deafness, Autism, Congential Heart Disease | Microarray, Sequencing of GJB2 and GJB6 | 3 | Yes | Partial | GJB2 (NM_004004.5) | AR | c.35delG (p.Gly12Valfs*2) (hom) | M/P | Partial genetic diagnosis. Category 1. Complex phenotype with Autosomal Recessive hearing loss caused by GJB2 variant. |
| 1063 | F | VACTREL associated with Klippel-Feil syndrome of cervical spine, Imperforate anus, Pierre Robin sequence, bilateral cleft lip and palate, Global developmental delay, agenesis of corpus callosum with colpocephaly, gastroesophageal reflux,tetralogy of fallot. | Microarray | 1 | No | No | - | - | - | - | Query VACTERL. No molecular diagnosis |
| 1064 | M | Hypoplastic thumbs, Hypoplastic R Kidney, L Hydronephrosis, vertebral segmentation aNomalies, congenital heartdefect, facial asymmetry | Microarray, chromosome breakage | 2 | No | No | - | - | - | - | Query VACTERL. No molecular diagnosis |
| 1065 | F | Limb reduction defects | Microarray, karyotype | 2 | No | No | - | - | - | - | Query Robert's syndrome. No molecular diagnosis |
| 1066 | M | Multiple parenchymal cavernomas, developmental delay and hydrocephalus | Microarray, Sequencing CCM2 | 2 | Yes | Yes | CCM2 (NM_031443.3) and 8q22.1del | AD | c.1054delG (p.Gly352Valfs*2) (het) and arr 8q22.1(97,145,564-98,301,541)x1 | SNV: P; CNV: DN | Complex phenotype with two genetic disorders. Category 1. Paternally inherited CCM2 pathogenic variant related to Cerebral cavernous malformation (CCM) diagnosis. Potential pharmacotherapy interventions for CCM. Category 2. Patient also has 8q22.1 de novo 1.16 Mb deletion. |
| 1067 | M | Mild Developmental delay, borderline macrosomic, ususual cranial sutures and increased wormina bones, unusual foramen magnum, hyperextensible | Microarray | 1 | No | No | - | - | - | - | No specific clinical or molecular diagnosis |
| 1068 | F | Coat hanger ribs, severe hypertonia, hand contractures | Microarray, UPD14 | 2 | Yes | No | - | - | - | - | Molecular diagnosis of UPD14 upon testing parental chromosomes |
| 1070 | F | bilateral multiple angiomyelomas (AMLs) (benign kidney tumor), query Tuberous Sclerosis, Von Wilebrand disease thought to be secondary to PDA with bleeding diathesis | Microarray, Sequencing TSC1 and TSC2 | 2 | No | partial | VWF (NM_000552.3) | AD | c.6187C>T (p.Pro2063Ser) (hom) | M/P | Partial genetic diagnosis. Category 1. Initially diagnosed with acquired Von Willebrand by hematology. However, molecular findings consistent with a genetic etiology. |
| 1071 | M | Seizures, hyrdocephalus, mid-brain astrocytoma | Microarray | 1 | No | No | - | - | - | - | No specific clinical or molecular diagnosis |
| 1072 | F | Intrauterine growth retardation-asymmetrical, hemihypertrophy, facial dysmorphism | Microarray, Methylation studies for RSS | 2 | Yes | No | - | - | - | - | Query Russell-Silver Syndrome. No molecular diagnosis |
| 1073 | F | Intellectual disability, dysmorphic features | Microarray | 1 | No | No | - | - | - | - | No specific clinical or molecular diagnosis |
| 1074 | M | Severe global developmental delay, dopa-responsive dystonia, spastic quadriplegic cerebral palsy | Microarray, Dystonia panel (Centogene) | 2 | No | No | - | - | - | - | Static spastic quadriplegic cerebral palsy. No molecular diagnosis |
| 1075 | F | Arthrogryposis, dysmorphic features, hypotonia, increased DTR, oral motor dysfunction | Microarray, Arthrogryposis Panel | 2 | No | No | - | - | - | - | No specific clinical or molecular diagnosis |
| 1076 | F | Global developmental delay, microcephaly, visual impairment | Microarray, microcephaly panel, Sequencing CLN6 | 3 | No | No | - | - | - | - | Batten disease. No molecular diagnosis |
| 1078 | M | Oculocutaneous Albinism, Intellectual disability, Obesity, ADHD | Microarray, karyotype, FISH 15q11.1, Methylation for PWS, Pigmentaion pannel (for OCA) | 3 | Yes | Yes | TYR (NM_000372.4) and MC4R (NM_005912.2) | AR | TYR:c.1118C>A (p.Thr373Lys) (het)/c.1205G>A (p.Arg402Gln) (het) and MC4R:c.307G>A (p.Val103Ile) | TYR; M/P | Complex phenotype with two related genetic disorders. Category 1. Oculocutaneous albinism type 1. Category 2. MC4R variant may be contribute to obesity |
| 1079 | M | Multiple congenital anomalies: left lung agenesis, left pulmonary artery agenesis, coronary artery fistula, right horseshoe kidney, coarse echotexture of liver, rudimentary gall bladder | Microarray | 1 | No | No | - | - | - | - | Possible Holt-Oram syndrome. No molecular diagnosis |
| 1080 | M | Congenital cataracts, perinatal stroke, global developmental delay | Microarray, Sequencing COL4A1 and COL4A2 | 3 | Yes | Yes | COL4A1 (NM_001845.4) | AD | c.2317G>A (p.Gly773Arg) (het) | DN | Category 1. MRI diagnosis of COL4A1-Related Disorder confirmed with sequence testing. Recommendations for surveillance by neurologist for disease related complications including neurological, ocular, cardiac, renal and Raynaud's phenomena. Also requires aggressive hypertension management to avoid strokes. |
| 1081 | M | Prenatal onset growth retardation, right limb defect, right nipple defect, right pectomal muscle hypoplasia, hypospadias, syndactyly | Microarray | 1 | No | No | - | - | - | - | Poland anomaly/syndrome. No molecular diagnosis |
| 1082 | M | Autism spectrum disorder, developmental delay, macrocephaly | Microarray, Fragile X, Fragile X E | 3 | No | No | - | - | - | - | No specific clinical or molecular diagnosis |
| 1083 | F | Short stature, Global developmental delay, facial dysmorphisms, panhypopituitarism | Microarray | 1 | No | No | - | - | - | - | No specific clinical or molecular diagnosis |
| 1084 | F | Hypotonia and brain abnormalities, increased axial CSF, brain hemorrhage, hypotonia, consanguinity | Microarray, Methylation for PWS, myotonic dystrophy panel | 3 | No | No | - | - | - | - | No specific clinical or molecular diagnosis |
| 1085 | M | Epilepsy, autism spectrum disorder, behavioral disorder | Microarray | 1 | No | No | - | - | - | - | No specific clinical or molecular diagnosis |
| 1086 | M | Congenital heart defects (tetralogy of Fallot, double-outlet right ventricle, ASD, VSD, pul stenosis), tracheal stenosis, hypoplastic thumb, radial hypoplasia | Microarray, Karyotype, 22q11.2 FISH, Sequencing and Dosage SALL1, TBX5, SALL4, chromosome breakage | 10 | No | No | - | - | - | - | Syndromic complex congenital heart malformation. No molecular diagnosis |
| 1088 | F | Increased body weight, umbilical hernia, protruding tongue, accessory nipple, positive family history | Microarray, CDKNIC sequencing, UPD11, 11p15.5 gene dosage, Methylation studies for chr11. | 5 | No | No | - | - | - | - | Unspecified overgrowth syndrome. No molecular diagnosis |
| 1089 | M | Coarse features, gingival hypertrophy, bilateral iris coloboma, severe hypoalbuminemia, edema, protein losing gastroenteropathy | Microarray, FISH chr12p13.2, Sequencing and dosage CHD7, Sequencing and Dosage GPC3, San Filipo Panel | 7 | No | Yes | PLVAP (NM_031310.1) | AR | c.1072C>T (p.Arg358*) (homo) | M/P | Category 3. Novel protein losing enteropathy disorder. |
| 1090 | F | Primary amenorrhea, delayed puberty, severe ID, seizures, blind, deaf, history of hydrocephalus and periventricular hemorrhage | Microarray, karyotype, Fragile X | 3 | Partial | Partial | Xp22.33-q21.32 and Xq21.32-q28 | AD | arr Xp22.33q21.32(60,701-91,873,757)x3, Xq21.32q28(91,877,172-155,174,078)x1 | DN | Partial genetic diagnosis. Category 1. Turner Syndrome but hydrocephalus is not explained by molecular finding. |
| 1091 | M | Chiari 1 malformation, coarctation of aorta and bicuspid aortic valve | Microarray | 1 | No | No | - | - | - | - | No specific clinical or molecular diagnosis |
| 1092 | M | Saggital synostosis, IUGR | Microarray | 1 | No | No | - | - | - | - | Craniosynostosis. No molecular diagnosis |
| 1093 | F | Spastic quadriplegia, global developmental delay, intractable epilepsy, failure to thrive, visual impairment, umblical hernia | Microarray, Sequencing and Dosage Infantile epilepsy panel (GeneDx) | 3 | No | Yes | NGLY1 (NM_001145294.1) and COG5 (NM_006348.3) | AR | c.517A>G p.Arg173Gly (hom) and c.1205C>T (p.Ser402Leu) (hom) | M/P | Complex phenotype with two related genetic disorders. Category 2. NGLY1 - Congenital disorder of glycosylation, type Iv. Category 1. COG5 - Congenital disorder of glycosylation, type IIi. |
| 1094 | F | History of learning difficulties, vertical nystagmus, amblyopia, cone-rod dystrophy (Diagnosed by electroretinogram) | Microarray, FragileX, Cone-rod dystrophy panel (Casey Eye Institute) | 3 | No | No | - | - | - | - | No specific clinical or molecular diagnosis |
| 1096 | M | Severe short stature, developmental delay | Microarray, Dosage and UPD for RSS | 3 | No | No | - | - | - | - | Methylation disorder or SHORT syndrome. No molecular diagnosis |
| 1097 | M | Obesity, facial dysmorphism | Microarray, obesity panel (Baylor), Sequencing LEP and LEPR, Methylation for PWS | 5 | No | No | - | - | - | - | Prader-Willi Syndrome. No molecular diagnosis |
| 1099 | F | Develomental delay, ptosis, brachydactyly | Microarray | 1 | No | No | - | - | - | - | No specific clinical or molecular diagnosis |
| 1100 | M | Myopia, learning disability, increased body weight | Microarray, Fragile X, Stickler syndrome comprehensive panel (CTGT), Achromatopsia gene panel (Casey eye institute) | 4 | No | No | - | - | - | - | Query Stickler Syndrome. No specific clinical or molecular diagnosis |
| 1101 | M | Profound global developmental delay, spastic quadriplegia, Acute rhabdomyolysis | Microarray, myopathy/Rhabdomyolysis panel (Baylor), Sequencing RYR1, X-linked MR associated with seizure panel | 5 | No | No | - | - | - | - | No specific clinical or molecular diagnosis |
| 1102 | M | Episodic hypotonia and developmental regression during febrile illness starting from 11 months of age | Microarray, telomeric FISH, ataxia/episodic ataxia NextGen panel (MNG ) | 3 | Yes | Yes | ATP1A3 (NM_152296.4) and 2p16.3 (NRXN1) | AD | arr 2p16.3 (51,021,507-51,358,841)x1 and c.2485G>A (p.Glu818Lys) (het) | both DN | Complex phenotype with two genetic disorders. Category 1. Pathogenic ATP1A3 variant associated with CAPOS Syndrome explains episodic hypotonia and regression history. Category 2. Patient also has pathogenic 2p16.3 337kb deletion overlapping NRXN1 gene associated with global developmental delay and Autism Spectrum disorder. |
| 1103 | F | Acquired microcephaly, global developmental delay, history of developmental regression associated with viral infection at 6 months age, myoclonic epilepsy | Microarray, telomere length analysis, clinical WES (Baylor) | 3 | Yes | Yes | VPS53 (NM_018289) | AR | c.1429C>T (p.Arg477*)/c.1716T>G (p.Ser572Arg) | M/P | Category 2. Pontocerebellar hypoplasia, type 2E. Older sibling with same clinical features, passed away at age 6 years, also diagnosed with the same disease afterwards. Followed in the metabolic genetics clinic for symptomatic treatment. |
| 1105 | F | Dysmorphic facial features, bicuspid aortic valve, reflux, developmental delay, joint pain | Microarray | 1 | No | No | - | - | - | - | No specific clinical or molecular diagnosis |
| 1106 | M | Global developmental delay, intractable epilepsy, ataxia, dystonia | Microarray, epilepsy panel sequencing and dosage (GeneDx) | 2 | No | No | - | - | - | - | Epileptic encephalopathy and movement disorder. No molecular diagnosis |
| 1107 | M | Prenatal onset short stature, congenital glaucoma, failure to thrive, microcephaly, prominence of trigones and occipital horns of lateral ventricles, hypercalcemia, coarse facial features, hypotonia, some stereotypic tapping hand movements | Microarray, FISH 7q11.23, Sequencing CASR | 3 | No | Yes | SMARCA2 (NM_003070) | AD | c.2639C>T (p.Thr880Ile) | DN | Category 1. Nicolaides-Baraitser syndrome. |
| 1108 | F | Seizure, failure to thrive/IUGR, dysmorphic facial features, large anterior fontanel, joint hypermobility blue sclerae, Global Developmental Delay, ASD, hypoglycemia, multiple fractures due to severe osteopenia, bilateral hip dysplasia, anaemia | Microarray, Epilepsy panel sequencing and dosage (GeneDx) | 3 | No | Yes | ALDH18A1 (NM_002860) | AR | c.1321C>T (p.Arg441*)/c.191G>A (p.Arg64His) | M/P | Category 2. Cutis laxa, type IIIA. Molecular diagnosis explains etiology of similar fatal disease in sibling and enables counselling regarding recurrence risks as well as definitive prenatal diagnosis. |
| 1112 | M | Seizures, Hearing and Visual impairment, Global Developmental delay, Central Nervous system malformations | Microarray | 1 | No | No | - | - | - | - | No specific clinical or molecular diagnosis |

^1^Sex: Male (M) and Female (F); ^2^IP: Inheritance Pattern; ^3^Origin of Transmission: De novo (DN), Paternal (P), Maternal (M), Not Available (N/A); ^4^All findings were relevant to Genetic counselling and were further split into categories based on clinical management: Category 1 (Disease-specific published management guidelines), Category 2 (Management based on case reports or known function of genes), and Category 3 (No management change)

## Supplementary Table 5: Whole genome sequencing coverage summary

| **Case ID** | **Coverage 1x** | **Coverage 5x** | **Coverage 10x** | **Coverage 40x** | **Mean coverage** |
| --- | --- | --- | --- | --- | --- |
| 1000 | 1.00 | 0.99 | 0.98 | 0.67 | 49.35 |
| 1001 | 1.00 | 1.00 | 0.99 | 0.75 | 52.62 |
| 1002 | 1.00 | 1.00 | 0.99 | 0.76 | 53.66 |
| 1003 | 1.00 | 1.00 | 0.99 | 0.78 | 53.37 |
| 1004 | 1.00 | 1.00 | 0.99 | 0.77 | 52.28 |
| 1005 | 1.00 | 1.00 | 0.99 | 0.77 | 53.30 |
| 1006 | 1.00 | 1.00 | 0.99 | 0.76 | 53.01 |
| 1007 | 1.00 | 1.00 | 0.99 | 0.80 | 54.34 |
| 1008 | 1.00 | 1.00 | 0.99 | 0.76 | 52.87 |
| 1009 | 1.00 | 1.00 | 0.99 | 0.78 | 54.04 |
| 1010 | 1.00 | 1.00 | 0.99 | 0.73 | 51.48 |
| 1011 | 1.00 | 1.00 | 0.99 | 0.79 | 53.92 |
| 1012 | 1.00 | 1.00 | 0.99 | 0.76 | 53.54 |
| 1013 | 1.00 | 1.00 | 0.99 | 0.76 | 53.01 |
| 1014 | 1.00 | 1.00 | 0.99 | 0.78 | 53.21 |
| 1015 | 1.00 | 1.00 | 0.99 | 0.74 | 52.05 |
| 1016 | 1.00 | 1.00 | 0.99 | 0.78 | 52.67 |
| 1018 | 1.00 | 1.00 | 0.99 | 0.77 | 54.07 |
| 1019 | 1.00 | 1.00 | 0.99 | 0.75 | 53.34 |
| 1020 | 1.00 | 1.00 | 0.99 | 0.70 | 50.50 |
| 1021 | 1.00 | 1.00 | 0.99 | 0.75 | 52.52 |
| 1022 | 1.00 | 1.00 | 0.99 | 0.75 | 52.87 |
| 1023 | 1.00 | 1.00 | 0.99 | 0.73 | 51.71 |
| 1024 | 1.00 | 0.99 | 0.99 | 0.69 | 49.43 |
| 1026 | 1.00 | 1.00 | 0.99 | 0.71 | 51.07 |
| 1027 | 1.00 | 1.00 | 0.99 | 0.76 | 52.35 |
| 1028 | 1.00 | 0.99 | 0.99 | 0.66 | 48.60 |
| 1029 | 1.00 | 1.00 | 0.99 | 0.79 | 54.66 |
| 1030 | 1.00 | 0.99 | 0.99 | 0.69 | 49.90 |
| 1031 | 1.00 | 1.00 | 0.99 | 0.71 | 51.03 |
| 1032 | 1.00 | 1.00 | 0.99 | 0.70 | 50.10 |
| 1034 | 1.00 | 1.00 | 0.99 | 0.78 | 53.36 |
| 1035 | 1.00 | 0.99 | 0.99 | 0.70 | 50.31 |
| 1036 | 1.00 | 0.99 | 0.99 | 0.67 | 49.24 |
| 1038 | 1.00 | 0.99 | 0.99 | 0.69 | 49.86 |
| 1039 | 1.00 | 0.99 | 0.99 | 0.70 | 50.75 |
| 1044 | 1.00 | 0.99 | 0.99 | 0.68 | 49.79 |
| 1045 | 1.00 | 0.99 | 0.99 | 0.67 | 49.40 |
| 1047 | 1.00 | 1.00 | 0.99 | 0.76 | 52.78 |
| 1050 | 1.00 | 1.00 | 0.99 | 0.73 | 50.90 |
| 1052 | 1.00 | 0.99 | 0.99 | 0.68 | 49.78 |
| 1055 | 1.00 | 1.00 | 0.99 | 0.71 | 50.26 |
| 1057 | 1.00 | 1.00 | 0.99 | 0.77 | 52.99 |
| 1058 | 1.00 | 1.00 | 0.99 | 0.74 | 52.44 |
| 1059 | 1.00 | 1.00 | 0.99 | 0.75 | 52.90 |
| 1061 | 1.00 | 1.00 | 0.99 | 0.79 | 53.62 |
| 1062 | 1.00 | 1.00 | 0.99 | 0.75 | 53.18 |
| 1066 | 1.00 | 0.99 | 0.99 | 0.56 | 43.34 |
| 1067 | 1.00 | 0.99 | 0.98 | 0.55 | 42.99 |
| 1068 | 1.00 | 1.00 | 0.99 | 0.63 | 45.84 |
| 1076 | 1.00 | 1.00 | 0.99 | 0.67 | 47.74 |
| 1078 | 1.00 | 0.99 | 0.99 | 0.66 | 47.80 |
| 1081 | 1.00 | 0.99 | 0.99 | 0.62 | 45.58 |
| 1082 | 1.00 | 0.99 | 0.99 | 0.64 | 46.10 |
| 1049 | 1.00 | 1.00 | 0.99 | 0.71 | 50.23 |
| 1072 | 1.00 | 1.00 | 0.99 | 0.66 | 47.17 |
| 1046 | 1.00 | 0.99 | 0.99 | 0.68 | 49.38 |
| 1051 | 1.00 | 1.00 | 0.99 | 0.72 | 49.81 |
| 1060 | 1.00 | 1.00 | 0.99 | 0.71 | 50.56 |
| 1064 | 1.00 | 0.99 | 0.99 | 0.59 | 44.15 |
| 1073 | 1.00 | 0.99 | 0.99 | 0.62 | 45.72 |
| 1079 | 1.00 | 0.99 | 0.99 | 0.71 | 50.63 |
| 1080 | 1.00 | 0.99 | 0.99 | 0.65 | 47.29 |
| 1040 | 1.00 | 0.99 | 0.99 | 0.67 | 48.84 |
| 1043 | 1.00 | 0.99 | 0.99 | 0.69 | 50.06 |
| 1053 | 1.00 | 1.00 | 0.99 | 0.68 | 48.53 |
| 1084 | 1.00 | 1.00 | 0.99 | 0.79 | 53.77 |
| 1085 | 1.00 | 1.00 | 0.99 | 0.77 | 53.50 |
| 1089 | 1.00 | 1.00 | 0.99 | 0.75 | 52.94 |
| 1090 | 1.00 | 1.00 | 0.99 | 0.78 | 54.24 |
| 1096 | 1.00 | 1.00 | 0.99 | 0.78 | 54.78 |
| 1042 | 1.00 | 0.99 | 0.99 | 0.67 | 48.64 |
| 1063 | 1.00 | 1.00 | 0.99 | 0.74 | 51.23 |
| 1070 | 1.00 | 1.00 | 0.99 | 0.66 | 47.15 |
| 1083 | 1.00 | 1.00 | 0.99 | 0.80 | 54.26 |
| 1086 | 1.00 | 1.00 | 0.99 | 0.78 | 54.78 |
| 1088 | 1.00 | 1.00 | 0.99 | 0.79 | 54.41 |
| 1093 | 1.00 | 1.00 | 0.99 | 0.80 | 54.53 |
| 1097 | 1.00 | 1.00 | 0.99 | 0.77 | 54.49 |
| 1100 | 1.00 | 1.00 | 0.99 | 0.76 | 53.92 |
| 1025 | 1.00 | 1.00 | 0.99 | 0.83 | 56.27 |
| 1056 | 1.00 | 1.00 | 0.99 | 0.77 | 54.52 |
| 1065 | 1.00 | 1.00 | 0.99 | 0.79 | 53.80 |
| 1074 | 1.00 | 1.00 | 0.99 | 0.76 | 53.70 |
| 1091 | 1.00 | 1.00 | 0.99 | 0.76 | 53.97 |
| 1092 | 1.00 | 1.00 | 0.99 | 0.76 | 53.29 |
| 1071 | 1.00 | 0.99 | 0.99 | 0.66 | 47.47 |
| 1099 | 1.00 | 1.00 | 0.99 | 0.80 | 54.99 |
| 1102 | 1.00 | 1.00 | 0.99 | 0.77 | 54.63 |
| 1108 | 1.00 | 1.00 | 0.99 | 0.82 | 55.92 |
| 1112 | 1.00 | 1.00 | 0.99 | 0.80 | 55.88 |
| 1048 | 1.00 | 0.99 | 0.99 | 0.70 | 50.54 |
| 1041 | 1.00 | 1.00 | 0.99 | 0.81 | 55.50 |
| 1075 | 1.00 | 1.00 | 0.99 | 0.80 | 54.39 |
| 1094 | 1.00 | 1.00 | 0.99 | 0.83 | 56.10 |
| 1105 | 1.00 | 1.00 | 0.99 | 0.83 | 56.59 |
| 1106 | 1.00 | 1.00 | 0.99 | 0.80 | 56.52 |
| 1103 | 1.00 | 1.00 | 0.99 | 0.81 | 54.97 |
| 1107 | 1.00 | 1.00 | 0.99 | 0.80 | 54.42 |
| 1101 | 1.00 | 1.00 | 0.99 | 0.79 | 55.20 |
| ***Average*** | ***0.998*** | ***0.995*** | ***0.990*** | ***0.734*** | ***51.76*** |
| ***Median*** | ***1.00*** | ***1.00*** | ***0.99*** | ***0.75*** | ***52.72*** |

## Supplementary Table 6: Whole genome sequencing variant summary

|  | **Mean** | **Median** | **Range** |
| --- | --- | --- | --- |
| **All Sequence Level variants** | 3,542,152 | 3,489,667 | 3,338,264 – 4,307,846 |
| **All Rare Variants <5%** | 157,849 | 135,451 | 121,746 - 441,209 |
| **Exonic Variants** | 20,014 | 19,718 | 18,400 - 24,594 |
| **Rare Variants Coding and predicted Damaging** | 498 | 462 | 374- 1,011 |
| **Potential Compound Heterozygous** | 38 | 31 | 13-140 |
| **Autosomal Dominant** | 22 | 21 | 10-51 |
| **Homozygous** | 5 | 3 | 0-27 |
| **X-Linked** | 2 | 1 | 0-10 |

## Supplementary Table 7: Whole genome sequencing CNV and SV summary

|  | ***All CNVs*** | | | ***Rare filtered variants^1^*** | | |
| --- | --- | --- | --- | --- | --- | --- |
|  | **CMA** | **WGS RD** | **WGS PE** | **CMA** | **WGS RD** | **WGS PE** |
| **Total (n=100)** | 578 | 24,809 | 160,428 | 165 | 1268 | 6578 |
| **#/sample** | 5.78 | 248.1 | 1,604 | 1.6 | 12.7 | 6.6 |
| **Mean size (bp)** | 652,397 | 29,364 | 1,886 | 1,218,953 | 114,123 | 4,189 |
| **Median size (bp)** | 119,050 | 10,000 | 495 | 129,777 | 12,000 | 1,135 |
| **Size range** | 355- 91,813,057 | 2,000-6,718,001 | 52- 679,226 | 355- 91,813,057 | 2,000-6,718,001 | 52- 679,226 |
| **Overlapping Exonic (%total)** | 415 (72%) | 6,888 (28%) | 3200 (2%) | 103 (62%) | 352 (28%) | 312 (4.7%) |
| **Overlapping OMIM morbid genes (%total)** | 93 (16%) | 1228 (5%) | 290 (0.2%) | 38 (23%) | 110 (8.7%) | 51 (0.8%) |

^1^Less than 3% frequency and <70% overlap with segmental duplications. RD= Read Depth Method; PE=Paired End Method.

## Supplementary Table 8: Illustrative case examples and impact on clinical management

| **Case ID** | **Phenotype and Genotype description** |
| --- | --- |
| 1009 | Presented with developmental delay, short stature, and metaphyseal dysplasia and over the course of the study was tested for Prader Willi syndrome, Fragile X, Noonan Syndrome, 22q11.2 dosage, and 15q11.2 dosage (Supplemental Table 4). All investigations were negative and CMA did not detect any clinically significant CNVs. Through WGS, we identified a homozygous 2bp deletion in exon 7 of *LARP7* (c.755_757del: p.Arg253Ile*6) causing a frameshift and premature stop codon. Loss of function mutations in *LARP7* are known to cause Alazami syndrome (OMIM#615071), which is characterized by facial dysmorphism, intellectual disability, and primordial dwarfism^1^. Being a very rare disorder with only a few cases described in the literature, this diagnosis would not have been considered by the referring clinician but upon re-examination of the phenotype, the diagnosis of Alazami syndrome was confirmed. |
| 1050 | Referred with a diagnosis of Juvenile Myelomonocytic Leukemia (JMML) pulmonary stenosis, and dysmorphism. Genetic testing included a karyotype, targeted FISH for 22q11.2 deletion syndrome, CMA, and a NGS Noonan syndrome panel interrogating 14 known genes. All investigations were normal. WGS detected a 25bp deletion overlapping a splice site in the gene *CBL* that was not detected on the Noonan NGS panel and confirmed to be de novo via Sanger sequencing (Supplemental Table 4 and Supplemental Figure 6). Presumably the size and location of the deletion made it difficult to detect using an NGS panel relying on capture or enrichment. |
| 1089 | Referred for coarse features, gingival hypertrophy, bilateral iris coloboma, and hypoalbuminemia. Endoscopy at 2 months of age showed generalized edema and at 5 months of age the patient developed sepsis leading to organ failure and death. A wide range of conventional genetic testing was normal (Supplementary Table 4) but WGS revealed a homozygous stop mutation (c.1072C>T:p.Arg358*) in *PLVAP* (plasmalemma vesicle associated protein) that likely causes a novel and distinct form of protein losing enteropathy (PLE)^2^. The phenotype is characterized by hypoproteinemia, hypoalbuminemia, and hypertriglyceridemia with a *Plvap* knockout mouse demonstrating a nearly identical phenotype demonstrating a critical role of *PLVAP* in endothelial barrier function^3^. As with WES, one of the advantages of an unbiased genetic test like WGS is the opportunity for the discovery of novel genes associated with human phenotypes. |
| 1102 | Presented with episodic hypotonia and developmental regression during febrile illness starting from 11 months of age. Genetic investigations included Microarray, sub-telomeric FISH, and an Ataxia/Episodic Ataxia Disorders panel (Medical Neurogenetics) consisting of 113 genes. Both the microarray and WGS testing revealed a pathogenic 300kb *de novo* deletion at 2p16.3 overlapping the NRXN1 gene that likely explains the global developmental delay in this patient. However, this CNV did not explain the episodic hypotonia and a heterozygous change in *ATP1A3* (c.2485G>A:p.Glu818Lys) was detected in the WGS that was also found to be *de novo* upon testing the parents. Mutations in *ATP1A3* are associated with CAPOS syndrome (OMIM**#**601338) and characterized by infant or child-hood onset of recurrent episodic regression with weakness, hypotonia and encephalopathy, particularly in the context of febrile illness. This is followed by permanent motor disturbances such as Ataxia or dystonia (movement disorder). The detection of two different pathogenic variants, one CNV and one SNV, illustrates the advantage of using WGS as a single genomic screen in complex phenotypes. |

## Supplementary Table 9: Clinically relevant exonic deletions

| **CaseID** | **Sex^1^** | **Size (Kb)** | **Zygosity** | **Gene** | **WGS Structural Variant** | **Disorder** | **Inheritance Pattern** |
| --- | --- | --- | --- | --- | --- | --- | --- |
| 1001 | M | 5.589 | Het | *FANCC* | Chr9:98006630- 98012219 | Fanconi Anemia, Complementation Group C | Autosomal Recessive |
| 1003 | F | 0.967 | Het | *CLN3* | ^2^Chr16:28497285-28498251 | Neuronal Ceroid lipofuscinosis 3 | Autosomal Recessive |
| 1019 | M | 5.014 | Het | *ANO5* | Chr11:22292790-22297803 | Gnathodiaphyseal dysplasia, Miyoshi muscular dystrophy 3, Muscular dystrophy, limb-girdle, type 2L | Autosomal Dominant /Autosomal Recessive |
| 1060 | M | 3.997 | Het | *NDUFB9* | Chr8:125,556,428-125,560,424 | Mitochondrial Complex 1 Deficiency | Autosomal Recessive |
| 1067 | M | 0.539 | Het | *GAA* | Chr17:78091657-78092195 | Glycogen storage disease II | Autosomal Recessive |
| 1082 | M | 2.567 | Het | *BFSP1* | Chr20:17479641-17482207 | Autosomal Recessive Cataract 33, Cortical | Autosomal Recessive |

^1^Sex: Male (M) and Female (F); ^2^Common deletion found in the vast majority of cases of Neuronal Ceroid lipofuscinosis 3 in patients of Finnish descent.

# Supplementary Figures


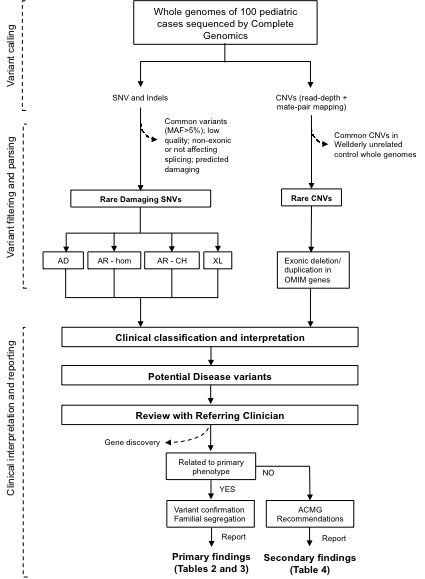


## Supplementary Figure 1: Overview of WGS Analysis

We built a systematic pipeline to prioritize both sequence level (SNVs and Indels) and CNVs of clinical significance (see supplemental text above for details). SNV=Single Nucleotide Variant. CNV=Copy Number Variant. AD=Autosomal Dominant. AR-hom=Autosomal Recessive Homozygous. AR-CH=Autosomal Recessive Compound Heterozygous. XL=X-linked.

**
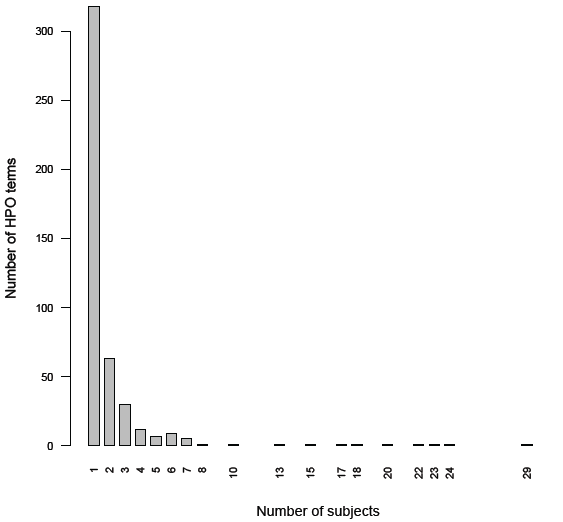
**

## Supplementary Figure 2: Histogram of frequency of HPO terms used in the cohort

The most common HPO term was ‘Developmental Delay’ and was used in 29 cases. The vast majority of HPO terms were used in only one subject.


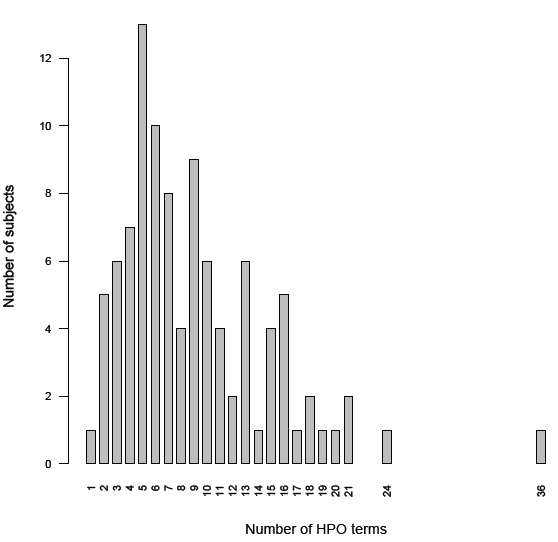


## Supplementary Figure 3: Histogram of number of HPO terms used to describe phenotypes

The number of phenotypes used to describe individuals ranged from 1-36. The mode, or most frequent number of terms used to describe a case was five.


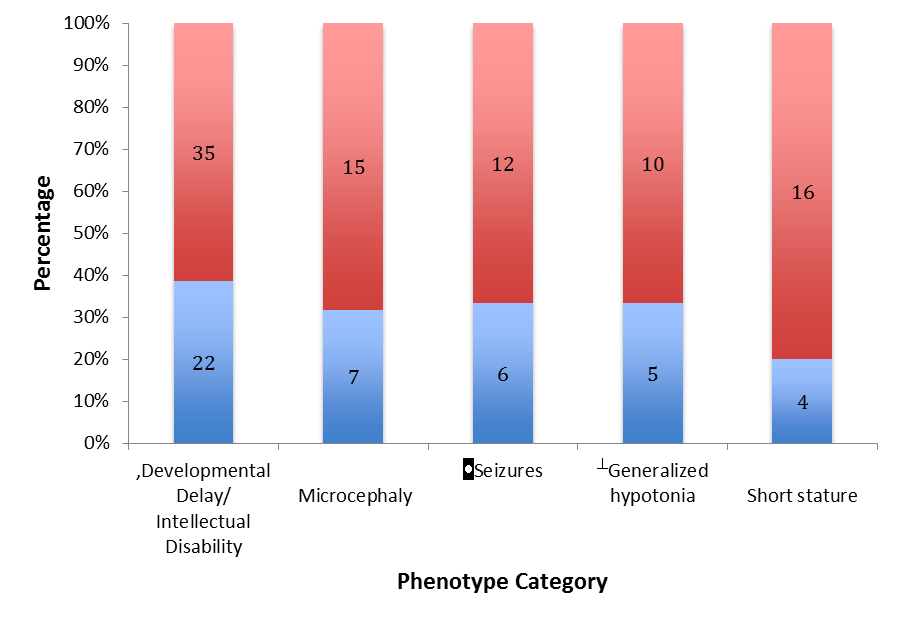


## Supplementary Figure 4: Stacked histogram of relative diagnostic rate across the common phenotypes in the cohort

Absolute numbers are shown for those with a genetic diagnosis (blue) and those that remained undiagnosed (red).


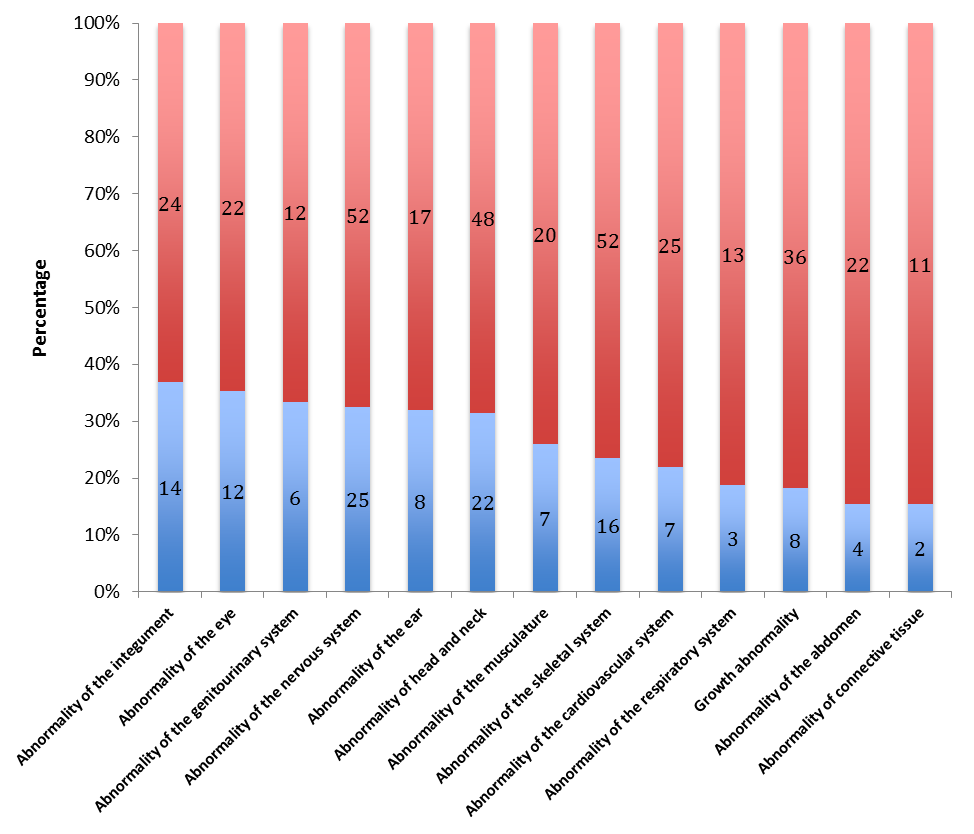


## Supplementary Figure 5: Stacked histogram of relative diagnostic rate across major HPO terms

Absolute numbers are shown for those with a genetic diagnosis (blue) and those that remained undiagnosed (red).


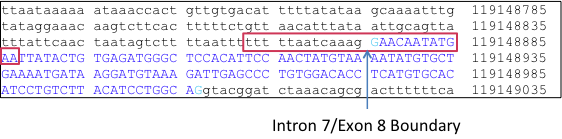


## Supplementary Figure 6: Deletion at intron-exon boundary in *CBS* gene

25bp deletion (red box) at intron exon boundary in the *CBL* detected through WGS in Case 1050. Lower case sequence depicts the intronic sequence and blue upper case depicts sequence from exon 8. Patient was referred with a diagnosis of Juvenile Myelomonocytic Leukemia (JMML).


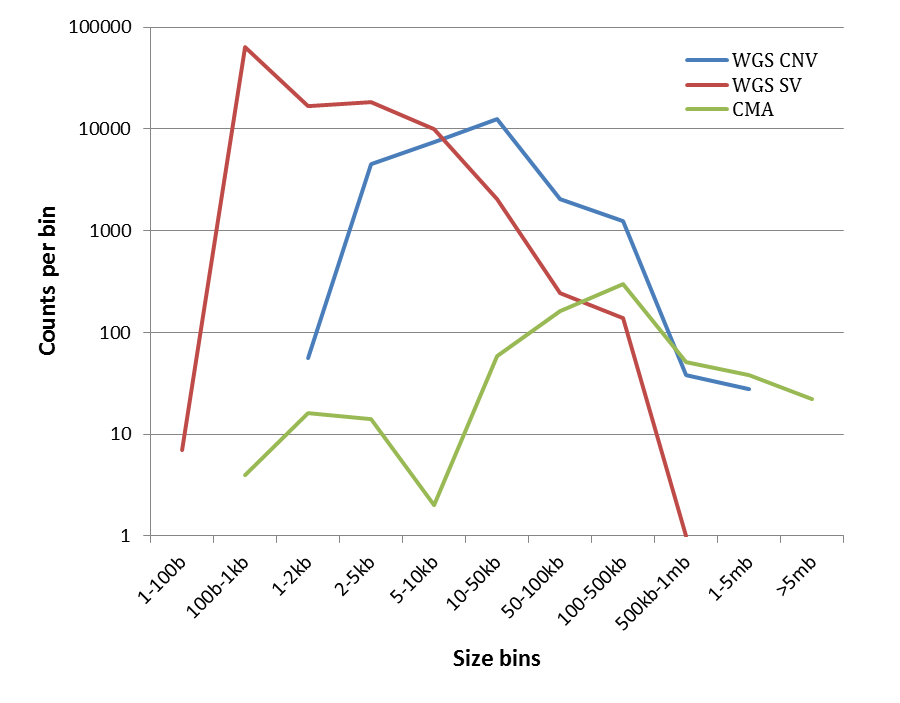


## Supplementary Figure 7: Binned Copy Number count in cohort using different detection methods

Cumulative Counts of CNVs detected per size bin across WGS paired end method (red; WGS SV), WGS read depth method (blue; WGS CNV) and Clinical Chromosomal Microarray (green, CMA)


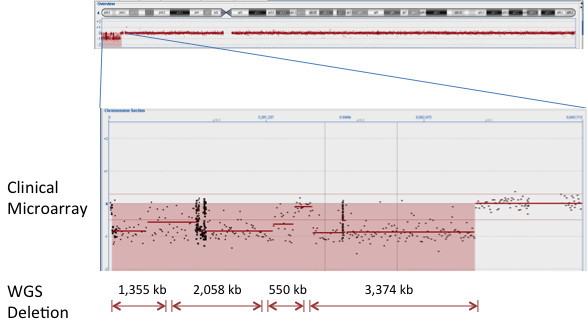


## Supplementary Figure 8: *De novo* 7.6 Mb deletion at 4p16.3-p16.1

Top panel shows location of terminal deletion with respect to chromosome, the enlarged version shows the deletion (red box) overlaid on the raw log2 probe plot. Bottom panel indicates the four CNV segments detected through the read depth method of the WGS.


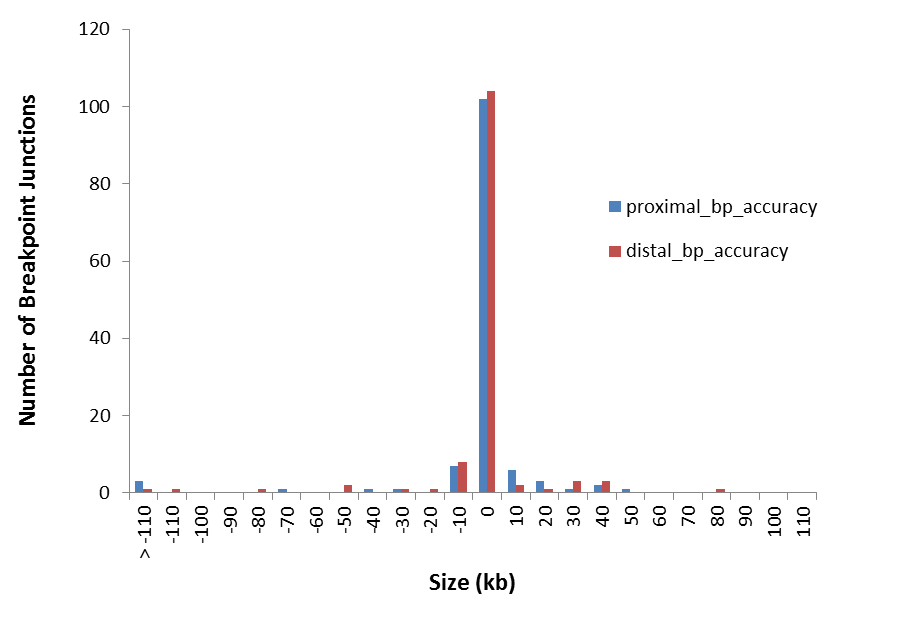


## Supplementary Figure 9: Beakpoint concordance of CNVs called in WGS and CMA

Histogram of breakpoint concordance for 139 CNVs detected by the WGS read depth method and Chromosomal Microarray analysis (CMA). Differences within CMA probe error were measured for the proximal (blue) and distal (red) breakpoints. 87% of the WGS breakpoints are within 10 kb of the CMA.

# Supplementary References:

1. Alazami AM, Al-Owain M, Alzahrani F, et al. Loss of function mutation in LARP7, chaperone of 7SK ncRNA, causes a syndrome of facial dysmorphism, intellectual disability, and primordial dwarfism. *Human mutation.* Oct 2012;33(10):1429-1434.

2. Elkadri A, Thoeni C, Deharvengt SJ, et al. Mutations in Plasmalemma Vesicle Associated Protein Result in Sieving Protein-Losing Enteropathy Characterized by Hypoproteinemia, Hypoalbuminemia, and Hypertriglyceridemia. *Cellular and molecular gastroenterology and hepatology.* Jul 2015;1(4):381-394 e387.

3. Stan RV, Tse D, Deharvengt SJ, et al. The diaphragms of fenestrated endothelia: gatekeepers of vascular permeability and blood composition. *Developmental cell.* Dec 11 2012;23(6):1203-1218.
